# Maternal bioactive lipids during pregnancy and early childhood neurodevelopment and behavior

**DOI:** 10.1038/s41390-025-04465-4

**Published:** 2025-10-08

**Authors:** Seonyoung Park, Megan L. Woodbury, Sung Kyun Park, Bhramar Mukherjee, Wei Hao, Lixia Zeng, Subramaniam Pennathur, Gredia Huerta Montañez, Zaira Rosario-Pabón, Carmen M. Vélez-Vega, José F. Cordero, Akram Alshawabkeh, Deborah J. Watkins, John D. Meeker

**Affiliations:** 1 Department of Environmental Health Sciences, University of Michigan School of Public Health, Ann Arbor, MI, USA.; 2 Department of Civil and Environmental Engineering, Northeastern University, Boston, MA, USA.; 3 Department of Epidemiology, University of Michigan School of Public Health, Ann Arbor, MI, USA.; 4 Department of Biostatistics, Yale University School of Public Health, New Haven, CT, USA.; 5 Department of Biostatistics, University of Michigan School of Public Health, Ann Arbor, MI, USA.; 6 Department of Internal Medicine, University of Michigan, Ann Arbor, MI, USA.; 7 Department of Molecular and Integrative Physiology, University of Michigan, Ann Arbor, MI, USA.; 8 Department of Social Sciences, UPR Medical Sciences Campus, University of Puerto Rico Graduate School of Public Health, San Juan, Puerto Rico.; 9 Department of Epidemiology and Biostatistics, University of Georgia, Athens, GA, USA.

## Abstract

**BACKGROUND:**

Maternal immune activation, including inflammation and oxidative stress during pregnancy, has been linked to child neurodevelopmental problems (NDP) and emotional and behavioral problems (EBP). Polyunsaturated fatty acids (PUFAs) and their oxidized metabolites (oxylipins) are important bioactive lipids that modulate immune responses, inflammation, and oxidative stress. However, their roles in child NDP and EBP remain unclear.

**METHODS:**

This study included 259 mother-child pairs from the PROTECT birth cohort in Puerto Rico. Maternal plasma samples collected around 26 weeks’ gestation were analyzed for bioactive lipid levels using high-performance liquid chromatography–tandem mass spectrometry. Child NDP and EBP were assessed at ages 1–3 using the Battelle Developmental Index, 2nd edition (BDI-2) and the Child Behavioral Checklist for ages 1.5–5 (CBCL/1.5–5). We evaluated associations between maternal bioactive lipid levels and child NDP or EBP outcomes and conducted sex-stratified analyses to examine effect modification by child sex. As a sensitivity analysis, we restricted analyses to term births to evaluate the associations independent of potential effects of preterm birth.

**RESULTS:**

Altered maternal bioactive lipid concentrations were associated with poorer neurodevelopmental and emotional/behavioral outcomes in children aged 1–3 years. The associations were modified by child sex and preterm birth status.

**CONCLUSION:**

These findings underscore the need for further research into bioactive-lipid-related maternal mechanisms that may influence early childhood neurodevelopment and behavior.

## INTRODUCTION

Child neurodevelopmental problems (NDP) encompass challenges in cognitive, adaptive, language, communication, motor, and social-emotional skills.^1^ NDP often coexist with childhood emotional and behavioral problems (EBP), which are classified into internalizing problems (such as depression, anxiety, and somatic complaints) and externalizing problems (such as aggression, inattention/hyperactivity, and oppositional/conduct issues).^2^ These challenges are often observed alongside neurodevelopmental disorders, including autism spectrum disorder (ASD) and attention-deficit/hyperactivity disorder (ADHD). Early-life NDP and EBP are significant because they can persist throughout life, contribute to productivity loss, and are linked to various mental disorders in adulthood.^3^ Recent studies have linked maternal immune activation involving inflammation and oxidative stress during pregnancy to child NDP and EBP.^4,5^ Maternal bioactive lipids during pregnancy are biologically plausible influencers of child NDP and EBP due to their crucial roles in the maternal immune response and inflammation.

Bioactive lipids are a diverse group of fats that are involved in inflammation and immune responses. This group includes the well-known omega-3 and omega-6 polyunsaturated fatty acids (PUFAs), such as eicosapentaenoic acid (EPA) and docosahexaenoic acid (DHA), along with their precursor α-linolenic acid (ALA), as well as arachidonic acid (AA) and its precursor linoleic acid (LA). When these PUFAs are oxidized, they form signaling molecules called oxylipins. Oxylipins are synthesized through three enzymatic pathways: cyclooxygenase (COX), lipoxygenase (LOX), and cytochrome p450 (CYP).^6^ PUFAs and oxylipins regulate essential physiological processes—including host defense, homeostatic maintenance, and inflammatory signaling.^7^ Although many oxylipins have been characterized as pro-inflammatory mediators,^8,9^ emerging evidence demonstrates that some oxylipins also possess anti-inflammatory and pro-resolving activities.^7,10^

Previous research has shown that oxylipins and their related enzymatic pathways are associated with neuroinflammation and the physiological processes of the central nervous system.^11,12^ For example, studies on ASD have identified elevated levels of Prostaglandin E2 in individuals with ASD, acting as a risk factor for neuroinflammation that contributes to various behavioral symptoms, including social impairment, depression, fever, and reduced food intake.^13^ Additionally, the LOX pathway, which metabolizes AA into oxylipins, plays a role in synaptic refinement and neuroplasticity. Recent animal studies using mouse models have demonstrated that altered expression of LOX-related genes may lead to motor deficits and behavioral abnormalities.^14,15^ Previous epidemiological studies have suggested an association between maternal PUFA intake during pregnancy and subclinical NDP and EBP in children.^16–18^ To date, limited numbers of studies have simultaneously evaluated an extensive panel of oxylipins alongside their parent PUFAs, or explored the co-occurrence of NDP and EBP within the same population.

In this study, we sought to examine the association between maternal bioactive lipid levels during pregnancy and NDP and/or EBP in children aged 1–3 years among mother-child pairs participating in the ongoing PROTECT birth cohort in Puerto Rico.

## METHODS

### Study participants

Participants in this study were part of the ongoing PROTECT study, a prospective cohort study based in Puerto Rico. Recruitment for the PROTECT cohort began in 2010, funded by the National Institute of Environmental Health Sciences Superfund Research Program, with the initial goal of examining the relationship between environmental contamination and preterm birth that has since expanded to include neurodevelopment of children born into the cohort. Women were enrolled at around 14 ± 2 weeks of pregnancy based on the following inclusion criteria: (1) age between 18 and 40 years; (2) residence in the Northern Karst region; (3) no use of oral contraceptives within three months prior to conception; (4) no significant obstetric or medical conditions reported; (5) no use of in vitro fertilization to conceive; and (6) no known medical complications. After obtaining informed consent, maternal blood samples were collected at the study visit around 26 weeks of gestation for bioactive lipid measurement. Maternal demographic and socioeconomic information was collected through a series of detailed questionnaires.^19^ Child neurodevelopmental and behavioral outcomes were measured from the PROTECT-born children by the Center for Research on Early Childhood Exposure and Development in Puerto Rico (CRECE)^20^ and as part of the Environmental Influences on Child Health Outcomes (ECHO) program.^21^ CRECE followed up children through 4 years of age from 2016 to 2019, then the monitoring continues through ECHO afterward. This study included 259 mother-child pairs with maternal bioactive lipid measurements during pregnancy and either child neurodevelopmental (*n* = 143) or emotional and behavioral outcome (*n* = 215) assessments between ages 1 and 3 years (Supplementary Fig. S1). This study was approved by the research and ethics committees of the University of Michigan School of Public Health, University of Puerto Rico, Northeastern University, and the University of Georgia.

### Bioactive lipids measurements

#### Bioactive lipid extraction from plasma.

A 100 μL sample of human plasma was mixed with 1 mL of 50 mM phosphate buffer at pH 7.4, supplemented with deuterated internal standards, as outlined previously.^22^ Bioactive lipids were isolated using Strata-X Polymeric SPE columns from Phenomenex, Inc., Torrance, CA. The columns were primed with 3 mL of pure methanol, balanced with 3 mL of water, and then filled with the plasma and internal standards mixture. After rinsing with 3 mL of 10% methanol, the bioactive lipids were collected with 2 mL of pure methanol and stored at −80 °C. All authentic bioactive lipids and their deuterated forms were purchased from Cayman Chemical (Ann Arbor, MI).

#### High-performance liquid chromatography-tandem mass spectrometry analysis.

Before testing, the eluants were evaporated under vacuum, reconstituted in 100 μL of solvent A (a mixture of water, acetonitrile, and acetic acid in a ratio of 60:40:0.02; v/v/v), filtered with 0.2 μm of PTFE Nano Filter Vial^®^ (Thomson Solutions, San Diego, CA) and then subjected for High-Performance Liquid Chromatography-Tandem Mass Spectrometry (HPLC/MS/MS) examination. Reverse-phase chromatography was applied using a Luna C18 LC column (100 Å, 3 μm, 150 × 2 mm; Phenomenex, Inc, Torrance, CA), using solvent A (a blend of water and acetonitrile in a 60:40 ratio, with 0.02% acetic acid) and solvent B (a mix of acetonitrile and isopropyl alcohol in a 50:50 ratio) on an Agilent 1200 LC system (Santa Clara, CA). For mass spectrometry, an Agilent 6490 Triple Quadrupole system with iFunnel technology was used. Bioactive lipids were identified based on unique fragmentation patterns and specific retention times without any isobaric interference in MS1. To quantify bioactive lipids, peak areas of the target analytes were compared with their respective isotopically labeled internal standards. The units used were μmol/L for primary compounds like AA, DHA, EPA, LA, and ALA, and nmol/L otherwise.

#### Quality control.

Sequential dilutions of each internal standard were conducted in duplicate to confirm linearity, determine the lower limit of detection (LOD), and estimate the coefficient of variation (CV) across different concentrations. In our study population, the majority of bioactive lipids had CVs below 20%, with additional details provided in our previous publication.^23^ Furthermore, pooled samples were analyzed at the start of each batch and after every 10 unknown samples during mass spectrometry to monitor potential drift in measurements over time and variations between batches, calculated as the CV.^24^ Most bioactive lipids were detectable above the LOD in over 75% of samples. For samples falling below the LOD, we used machine-read values when available to avoid skewing data with duplicate values, which could impact distribution and statistical calculations. This strategy aligns with approaches used in prior research.^25–28^ We excluded a negative concentration of Prostaglandin B2 from the analysis. Bioactive lipids were classified into four groups: (1) Parent compound PUFAs, including ALA, LA, AA, DHA, and EPA; and oxylipins derived from three enzymatic pathways—(2) COX, (3) CYP, and (4) LOX.

### Child neurodevelopmental assessments

#### Battelle developmental index-2 Spanish edition (BDI-2).

Child NDP was assessed using the Battelle Developmental Index-2 Spanish edition (BDI-2) at ages 1, 2, and 3 years. The BDI-2 evaluates age-specific neurodevelopmental functions across five domains: adaptive, cognitive, personal-social, communication, and motor skills.^29^ The BDI-2 total score combines information from all domains. For each domain and the total score, the BDI-2 provides a developmental quotient score (DQ score),^30^ which is norm-referenced with a mean of 100 and a standard deviation of 15. Lower DQ scores suggest poorer performance in that domain and potential developmental delays. We analyzed DQ scores for the five individual domains as well as the total score for children aged 1 to 3 years.

#### Child behavioral checklist for ages 1.5–5 (CBCL/1.5–5).

Child EBP were assessed using the Child Behavioral Checklist for ages 1.5–5 (CBCL/1.5–5), a psychometric tool used to evaluate EBP in preschool children.^31^ In our analysis, we included CBCL/1.5–5 outcomes measured between ages 1.5 and 3 years. This widely recognized tool asks primary caregivers to rate the frequency of 99 different behaviors or emotions of the child using a three-point scale: 0 (not true), 1 (somewhat/sometimes true), and 2 (very/often true), along with an open-ended 100th question.^32^ The CBCL/1.5–5 generates seven “syndrome” scale scores: Emotionally Reactive, Anxious/Depressed, Somatic Complaints, Withdrawn, Sleep Problems, Attention Problems, and Aggressive Behavior. These individual syndrome scores are then grouped into three “composite” scales: internalizing problems (combining scores from emotionally reactive, anxious/depressed, withdrawn, and somatic complaints), externalizing problems (aggregating scores from attention problems and aggressive behavior), and total problems (the sum of all scores, plus the highest score—either 1 or 2—from any additional issues noted in the open-ended 100th question). We included raw scores of the three composite scales for ages 1.5–3 years in our analyses. Higher scores indicate a greater number of EBPs. Raw scores were used instead of standardized T-scores as the T-scores are truncated at 50 and the CBCL/1.5–5 manual recommends using raw scores for research purposes.^31^

### Statistical analyses

Distribution of plasma bioactive lipids was assessed prior to analysis. Bioactive lipid concentrations were natural log-transformed to improve normality and included as continuous variables in the regression models. The associations between maternal bioactive lipid concentrations and BDI-2 DQ scores were examined using linear mixed effects models with subject-specific random intercepts to account for repeated outcome measures. Since BDI-2 DQ scores are normalized, they were included in the models without additional transformation. However, we utilized negative binomial regression models to examine the association between maternal bioactive lipid concentration and child CBCL/1.5–5 scores. This is because CBCL/1.5–5 scores are summed counts of behaviors, heavily right skewed, over-dispersed, and common transformation methods (i.e., logarithm and square root) did not improve distributions. This approach has been used in previous studies.^33–36^ We also included subject-specific random intercepts in the negative binomial regression models to account for repeated outcome measurements. All the regression coefficient estimates, $\overset{ˆ}{\beta }$, were converted to percent change per doubling of bioactive lipid concentration by using following formulas. For BDI-2, percent change was calculated as $\left[\left\{(ln2\;*\overset{ˆ}{\beta })/\text{median}\right\}\;*\;100\right]$, where ln2 is the natural logarithm of 2, $\overset{ˆ}{\beta }$ is the estimated coefficient from the linear mixed effects model, and the median is the median value of each BDI-2 DQ score. For CBCL/1.5–5, the percent change was calculated as $\left[\{exp(ln2^{*}\overset{ˆ}{\beta })-1\}\times 100\right]$, where $\overset{ˆ}{\beta }$ is the estimated coefficient from the negative binomial regression model.

Covariates included in the final model were selected based on a priori knowledge^37–39^ and aligned with previously published analyses from the PROTECT cohort.^40–43^ In addition, potential laboratory artifacts were evaluated. Our preliminary analyses, including batch as a covariate, indicated that batch did not appreciably impact the main effect (data not shown). The final models were adjusted for maternal age (continuous), maternal education (categorical; GED or less, some college, bachelor’s or higher), pre-pregnancy BMI (continuous), child age in months (continuous), and child sex. Additionally, we accounted for the influence of dietary fatty acid intake. The balance between omega-6 and omega-3 is known to play an important role in oxylipin production^7,44^ and the ratio of omega-6 to omega-3 fatty acids (N6:N3) in plasma is a well-validated indicator of dietary fatty acid intake.^44,45^ In addition, previous studies have shown that N6:N3 during pregnancy is associated with poorer child neurodevelopment in ages 6 months,^17^ and 2–3 years.^46^ Here we utilized the ratio of quantified PUFAs (i.e., ∑[LA + AA]/∑[DHA + EPA + ALA]) as a covariate and similar approach was used in a previous epidemiological research.^47^

We examined effect modification by child sex, based on prior evidence suggesting sex-specific vulnerability to child NDP and EBP.^48,49^ We first tested an interaction term between lipid levels and child sex. Based on the results, we presented sex-stratified findings to facilitate interpretation. As a sensitivity analysis, we excluded the N6:N3 covariate and compared the regression results with main models. Additionally, we also limited our analyses to term birth children (*N* = 130 for BDI-2 and *N* = 199 for CBCL/1.5–5) to examine the associations without the effect of preterm birth. Given the exploratory nature of this study and the limited sample size, we did not adjust for multiple comparisons.

Linear mixed-effects models were conducted using the “lme4” package (version 1.1–35.5), and negative binomial regression models were performed using the “glmmTMB” package (version 1.1.9) in R.

All statistical analyses were carried out using R version 4.0.5.

## RESULTS

### Participant characteristics

The demographic and health characteristics of the study participants (*N* = 143 mother-child pairs for BDI-2 analysis and *N* = 215 mother-child pairs for CBCL/1.5–5 analysis) are summarized in Table 1. In general, most women in the analysis were under the age 30 (61.6% in the BDI-2 sample and 61.9% in the CBCL/1.5–5 sample), had received a college degree (86% in the BDI-2 sample and 83.2% in the CBCL/1.5–5 sample), were married or cohabitating with a partner (85.3% in the BDI-2 sample and 87% in the CBCL/1.5–5 sample), were currently employed (65% in the BDI-2 sample and 62.3% in the CBCL/1.5–5 sample), had an annual household income under 30 k (51.1% in the BDI-2 sample and 53.9% in the CBCL/1.5–5 sample), had a pre-pregnancy BMI under 30 kg/m^2^ (75.6% in the BDI-2 sample and 73.5% in the CBCL/1.5–5 sample), had never consumed alcohol (53.1% in the BDI-2 sample and 60.5% in the CBCL/1.5–5 sample), and had <2 total pregnancies (76.3% in the BDI-2 sample and 74% in the CBCL/1.5–5 sample). Most children were born at term (≥37 weeks) both in the BDI-2 analysis (90.9%) and the CBCL/1.5–5 analysis (92.6%) samples, with a balanced distribution of child sex overall. For the BDI-2 analysis, out of 143 children, 31 (21.7%) had BDI-2 outcomes measured twice, and 9 children (6.3%) had BDI-2 scores measured at three different ages. For the CBCL/1.5–5 analysis, out of 215 children, 54 (25.1%) had CBCL/1.5–5 scores measured twice, and 12 children (5.6%) had scores measured at three different ages. The distribution of maternal bioactive lipid concentrations in the study population is presented in Table 2. The possible range and the distribution of BDI-2 domain scores and CBCL/1.5–5 composite scores in this study sample are presented in Table 3. Additionally, distributions of BDI-2 scores and CBCL/1.5–5 scores by participant characteristics are summarized in Supplementary Tables S1 and S2. In general, the outcome measures —BDI-2 domain DQ scores and CBCL/1.5–5 composite scores—are comparable across different demographic characteristics with only a few exceptions.

### Maternal bioactive lipid and BDI-2 outcomes

**Fig. 1:**
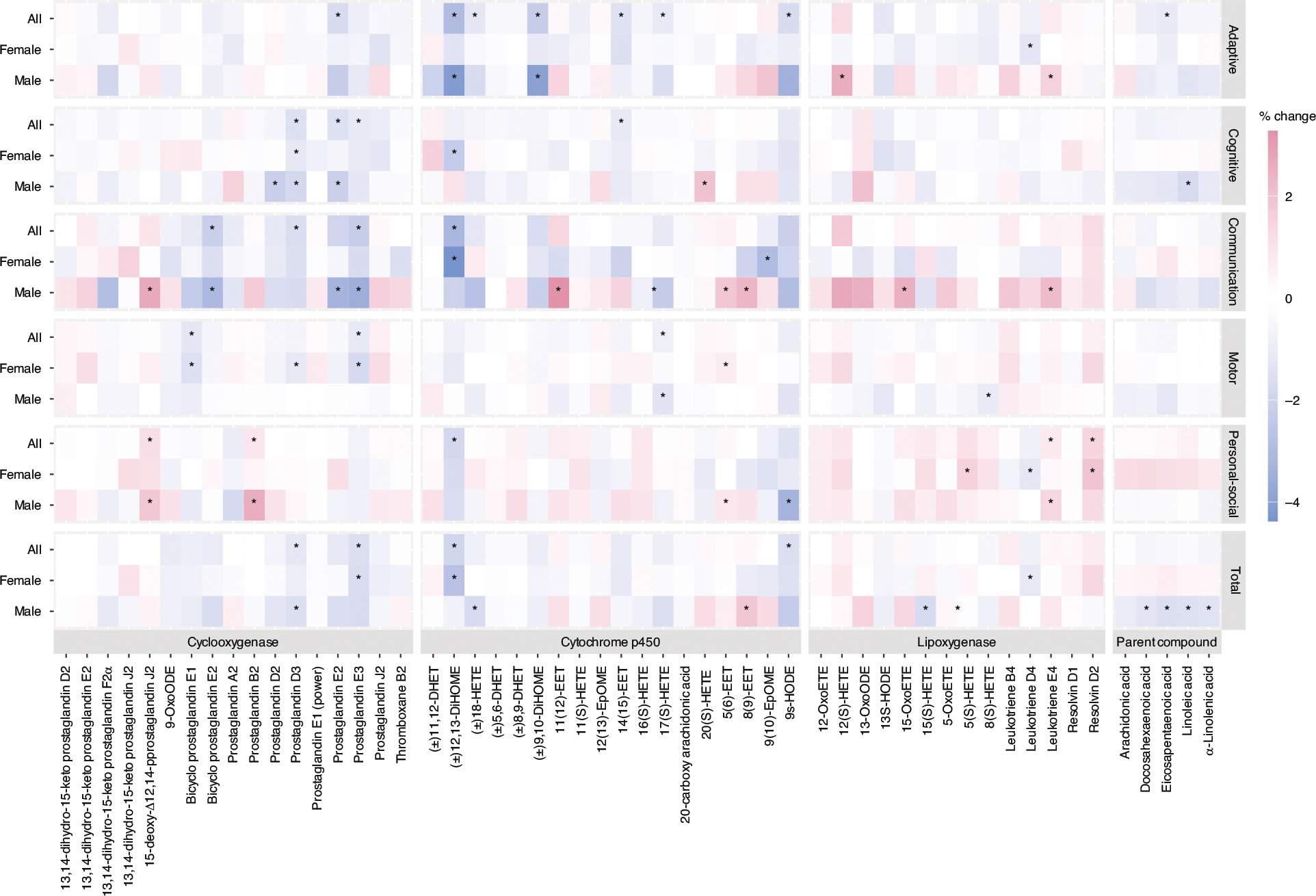
Percentage change in BDI-2 scores per doubling of maternal bioactive lipid concentrations, stratified by child sex. The number of participants and sample size for each group were as follows: *N*_all_ = 143 (192), *N*_Female_ = 77 (110), *N*_Male_ = 66 (82). Asterisk (*) indicates statistical significance (*p* < 0.05).

Child neurodevelopment between ages 1 and 3 years—across five domains assessed by the BDI-2 (Adaptive, Personal-Social, Communication, Motor, and Cognitive) and overall developmental status—was associated with maternal bioactive lipid levels during pregnancy (Fig. 1). Generally, we observed negative trends of associations, indicating poorer child neurodevelopmental status related to increased maternal bioactive lipid levels. For example, a doubling of Prostaglandin D3 and E3 concentrations from the COX pathway was associated with 1.22% (95% CI: −2.14, −0.3) and 1.59% (95% CI: −2.59, −0.59) lower BDI-2 Total scores, respectively. From the CYP pathway, a doubling of (±)12,13-DiHOME concentration was associated with a 1.96% lower BDI-2 Total score (95% CI: −3.67, −0.25), while 9s-HODE was associated with a 1.56% decrease per doubling (95% CI: −3.09, −0.04) in the Total score. Each domain showed associations with multiple oxylipins, varying by enzymatic pathway. Specifically, the Adaptive domain showed associations with multiple oxylipins from the CYP pathway, while the Cognitive and Communication domains were associated with oxylipins from the COX pathway. For example, the Adaptive domain was negatively associated with (±)12,13-DiHOME, (±)18-HETE, (±)9,10-DiHOME, 14(15)-EET, 17(S)-HETE, and 9s-HODE from the CYP pathway. However, lower Cognitive domain scores were observed in relation to higher concentrations of Prostaglandin D3, E2, and E3 from the COX pathway. Similarly, the Communication domain was inversely associated with Bicyclo Prostaglandin E2, Prostaglandin D3 and E3 concentrations (COX pathway). While most associations were negative, the Personal-Social domain showed positive associations with certain oxylipins, indicating that higher concentrations were linked to higher scores (reflecting better performance). For instance, a doubling of the 15-deoxy-Δ12,14-Prostaglandin J2 and Prostaglandin B2 concentrations from the COX pathway was associated with higher Personal-Social domain score by 1.18% (95% CI: 0.23, 2.12) and by 0.92% (95% CI: 0.01, 1.82), respectively. Moreover, a doubling of Leukotriene E4 and Resolvin D2 concentrations from the LOX pathway was associated with 0.77% (95% CI: 0.03, 1.51) and 1.46% (95% CI: 0.1, 2.82) higher scores in the personal-social domain, respectively. More detailed regression results are summarized in Supplementary Table S3.

In analyses stratified by child sex, we observed several child sex-specific differences in the associations between maternal oxylipin and parent compound PUFAs with child BDI-2 scores (Fig. 1). Notably, several associations were only observed among male children. For example, the Total BDI-2 score was associated with most parent compounds, except for AA, only among male children, while none of these associations were observed among female children. Similarly, associations with the Adaptive, Cognitive, and Communication domains were more pronounced among male children than female children. Specifically, a doubling of the 12(S)-HETE concentration from the LOX pathway was associated with a 2.59% increase in the Adaptive domain score (95% CI: 0.67, 4.51), and a doubling of the Prostaglandin D2 concentration from the COX pathway was associated with a 2.19% lower Cognitive score (95% CI: −4.05, −0.33) among male children. Additionally, several positive associations with the Communication domain were observed among male children in the stratified analysis but not in the overall analysis. These include associations of 15-deoxy-Δ12,14-Prostaglandin J2 (2.71% per doubling, 95% CI: 0.49, 4.92) from the COX pathway; 11(12)-EET (3.27% per doubling, 95% CI: 0.69, 5.84), 5(6)-EET (1.86% per doubling, 95% CI: 0.52, 3.2), and 8(9)-EET (2.34% per doubling, 95% CI: 0.08, 4.61) from the CYP pathway; and 15-OxoETE (2.54%/doubling, 95% CI: 0.38, 4.7) and Leukotriene E4 (2.24%/doubling, 95% CI: 0.48, 4.01) from the LOX pathway. In contrast, the Motor domain score was negatively associated with several oxylipins— Bicyclo Prostaglandin E1, Prostaglandin D3, and Prostaglandin E3—from COX pathway only among female children. Resolvin D2 from the LOX pathway showed a positive association with the Personal-Social domain in the overall group and female children, but not when limited to male children.

### Maternal bioactive lipid and CBCL/1.5–5 outcomes

**Fig. 2:**
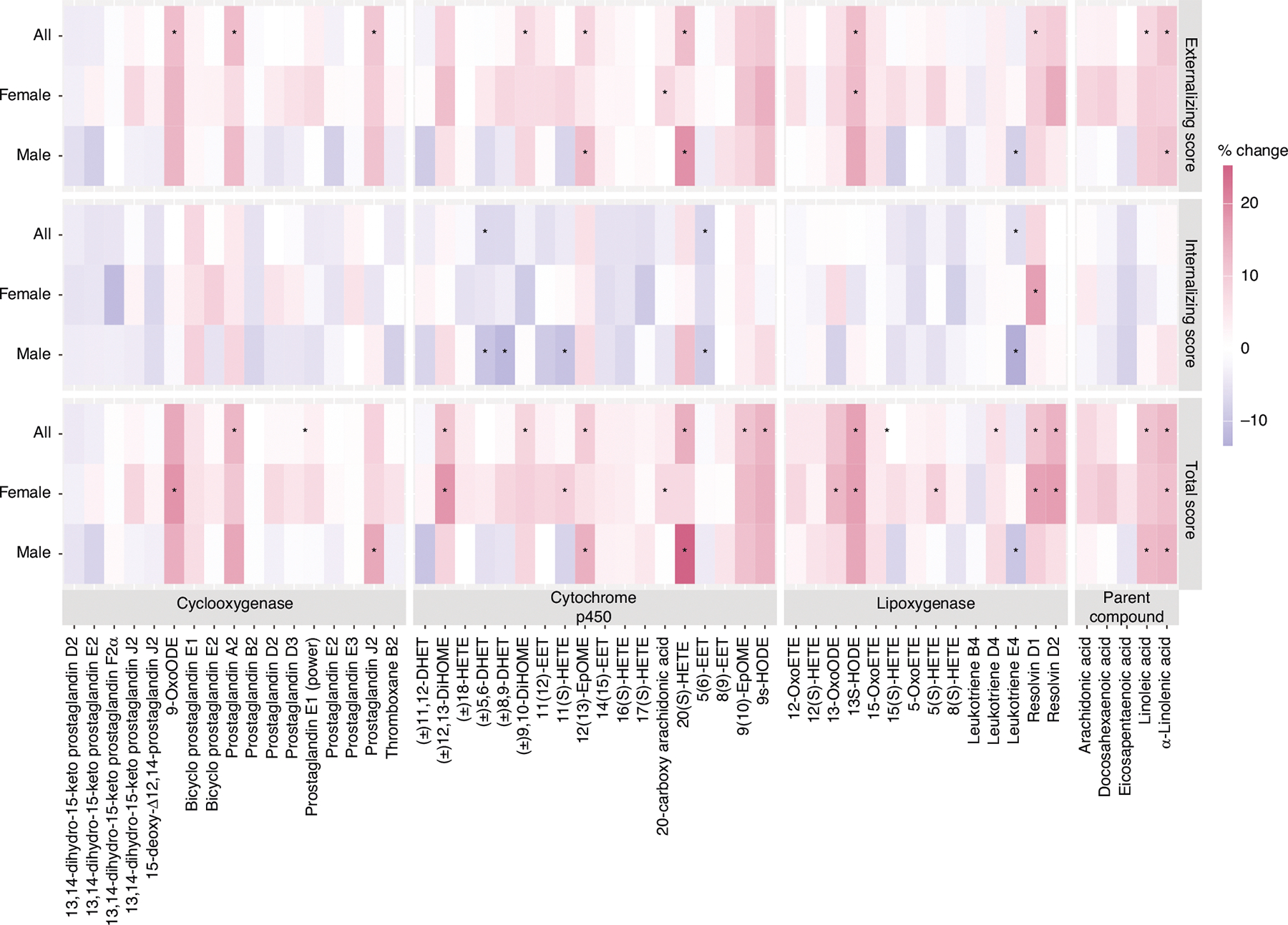
Percent change in CBCL/1.5–5 scores per doubling of maternal bioactive lipid concentrations, stratified by child sex. The number of participants and sample size for each group were as follows: *N*_all_ = 215 (293), *N*_Female_ = 110 (151), *N*_Male_ = 105 (142). Asterisk (*) indicates statistical significance (*p* < 0.05).

In our main analyses of CBCL/1.5–5 for which higher scores indicate more problematic symptoms, we observed positive trends of associations between maternal bioactive lipid concentrations with both the Externalizing composite score and the total score, while the Internalizing composite score was negatively associated (Fig. 2). Interestingly, several oxylipins from all three pathway groups as well as parent compound PUFAs were positively associated with both the Externalizing and Total scores. These included 9-OxoODE from the COX pathway, 12(13)-EpOME from the CYP pathway, 13S-HODE from the LOX pathway, and the parent compound LA. Specifically, a doubling of 9-OxoODE concentration was associated with 12.5% higher Externalizing score (95% CI: 0.9, 25.44) and 15.73% higher Total score (95% CI: 3.32, 29.63). Similarly, a doubling of 13S-HODE concentration was associated with 12.61% higher Externalizing score (95% CI: 2.31, 23.94) and 16.8% higher Total score (95% CI: 6.16, 28.52). On the other hand, the Internalizing score was negatively associated with (±)5,6-DHET (−6.86% per doubling, 95% CI: −12.4, −0.97) and 5(6)-EET (−7.43% per doubling, 95% CI: −11.95, −2.67) from the CYP pathway and Leukotriene E4 (−6.06% per doubling, 95%: −11.39, −0.41) from the LOX pathway, indicating that lower concentrations of these oxylipins are associated with more problematic symptoms.

From child-sex-stratified analyses, we observed several differences by child sex. Some of the positive associations with the Externalizing score and Total score were stronger among male children than female children. For instance, a doubling of 12(13)-EpOME concentration was associated with a 12.41% higher externalizing score (95% CI: 1.74, 24.19) and a 14.54% higher Total score (95% CI: 4.06, 26.08) among male children, but not among female children. Additionally, a negative trend in the associations of the Internalizing score was predominantly observed among male children, especially with oxylipins from the CYP pathway. For instance, a doubling of (±)8,9-DHET and 5 (6)-EET concentrations was associated with an 11.98% lower (95% CI: −21.38, −1.46) and an 8.53% lower (95%CI: −14.92, −1.67) in the Internalizing score among male children, respectively. However, there are also a few associations stronger among female children. For example, 20-hydroxyarachidonic acid (20-HETE) from the CYP pathway and 13-HODE from the LOX pathway showed positive associations with the Externalizing and Total scores among female children but not among male children. More detailed regression results are summarized in Supplementary Table S4.

### Sensitivity analyses results

We repeated our main modeling, excluding the N6:N3 covariate in the model and compared the results with the main analyses results to examine a potential confounding effect of dietary fatty acid consumption. Only a few associations differed after excluding N6:N3 in the model (Supplementary Figs. S2 and S3). Differences include the disappearance of associations of Bicyclo Prostaglandin E2 or 17(S)-HETE and the Motor domain from the BDI-2 analyses, and new associations of 9(10)-EpOME and 9s-HODE with the Externalizing score from the CBCL/1.5–5 analyses. However, in general, most associations of oxylipin and parent compound with BDI-2 or CBCL/1.5–5 scores were similar regardless of the inclusion of the dietary fatty acid consumption indicator in the model. We also repeated our main modeling by limiting the analysis to term birth children to examine the associations between bioactive lipids and child NDP and EBP outcomes independent of the effect of preterm birth. We noticed that several associations weakened after excluding preterm birth children from analyses. Specifically, the associations of Prostaglandin D3 (COX pathway), (±)18-HETE (CYP pathway), and parent compounds with the Total BDI-2 among male children were not observed when excluding preterm birth children. Similarly, several positive associations of oxylipins from the COX and the CYP pathways with the Externalizing and the Total CBCL/1.5–5 scores were no longer evident. Supplementary Figs. S4 and S5 summarized the results. These results suggest that preterm birth may contribute to the relationship between maternal bioactive lipid concentrations and child neurodevelopmental and behavioral outcomes.

## DISCUSSION

In this study, we observed several associations between maternal bioactive lipids and child neurodevelopmental outcomes between ages 1 and 3 years, as well as emotional and behavioral outcomes between ages 1.5 and 3 years. Analyses of BDI-2 outcomes generally showed negative associations across Adaptive, Personal-Social, Communication, Motor, and Cognitive domains, suggesting an increased risk of NDP associated with higher maternal bioactive lipid levels. In analyses using CBCL/1.5–5 outcomes, maternal bioactive lipid concentrations were positively associated with both the Externalizing composite score and the Total score, while negative associations were observed with the Internalizing composite score. These findings suggest that altered maternal bioactive lipid concentrations may be linked to increased risk of child EBP. In both the BDI-2 and CBCL/1.5–5 analyses, sex-stratified models indicated that the associations observed in the overall sample were primarily driven by male children. Sensitivity analyses further showed that several associations were attenuated after excluding children born preterm, suggesting that preterm birth may play a role in the observed relationships between maternal bioactive lipid concentrations and child NDP and EBP.

Previous studies have examined associations between maternal parent compound PUFAs and oxylipins with child neurodevelopmental or behavioral outcomes but generated inconclusive findings. For example, a Norwegian cohort study reported that higher maternal DHA levels during pregnancy (~28 weeks gestation) were positively associated with infants’ problem-solving abilities at 12 months of age.^50^ Liu et al. showed that maternal erythrocyte EPA measured between 20 and 28 weeks gestation was associated with a reduced risk of potential developmental delay in gross motor skills, while maternal AA was linked with higher risk of potential developmental delay in personal–social skills.^51^ However, Crozier et al. measured AA and DHA levels during early (~12 weeks) and late (~34 weeks) pregnancy and found no association between these fatty acids and children’s neurocognitive function at ages 4 or 6–7 years.^52^ Similarly, Kim et al.^17^ estimated maternal dietary fatty acid intake during pregnancy before 20 weeks of gestation using dietary interviews and examined its association with cognitive and motor development assessed by the Korean version of the Bayley Scales of Infant Development, Second Edition (BSID-II). They found no significant associations between PUFA intake (i.e., LA and ALA) and child cognitive or motor development at 6 months of age. Moreover, a recent study from the Generation R cohort in the Netherlands found that higher maternal plasma DHA concentrations around 20.5 weeks of gestation were associated with fewer EBPs at age 6, as assessed by the CBCL/1.5–5 using both parent and combined teacher/parent reports.^53^ In contrast, higher maternal plasma AA concentrations were associated with more problems, based on teacher and combined teacher/parent scores.^17,52,53^ To our knowledge, few studies have investigated the relationship between oxylipins and child neurodevelopmental or emotional and behavioral outcomes. Che et al.^54^ reported that compared to controls ASD boys (identified at ages 3, 5, and 7 years) had lower levels of maternal plasma 17-hydroxy-4,7,10,13,15,19-DHA measured between 17 and 21 weeks of gestation and leukotriene B4 in cord blood, while showing higher concentrations of AA in cord blood. In our analyses, only a few associations were observed between maternal PUFAs and child neurodevelopmental outcome (BDI-2), while most associations were led by oxylipins. For child emotional and behavioral outcomes (CBCL/1.5–5), associations were observed with PUFAs as well as oxylipins, however, the associations were prominent in LA or ALA unlike in the previous study. These discrepancies may be attributed to: (1) differences in the biological matrices where PUFAs or oxylipins were measured (e.g., plasma vs. serum vs. erythrocyte), (2) variation in the age at which child neurodevelopment and emotional and behavioral outcomes were assessed, and (3) the use of different assessment tools across studies. Additionally, due to the limited number of epidemiological studies that have examined a comprehensive panel of oxylipins alongside PUFAs in relation to child NDP or EBP, meaningful comparisons remain challenging. Future studies that employ harmonized measurement approaches for both maternal bioactive lipids and child neurodevelopmental and behavioral outcomes are warranted to enhance comparability and strengthen the evidence base.

Nonetheless, the findings from the current study offer valuable insights, as they may be understood in the context of emerging patterns. In particular, the observed associations appeared to differ according to the inflammation-related activity of the bioactive lipids. For example, we observed that several BDI-2 domain scores had negative associations with pro-inflammatory bioactive lipids, indicating worse performance related to elevated pro-inflammatory lipid concentration. In contrast, positive associations primarily appeared with anti-inflammatory bioactive lipids, which implies better performance related to higher anti-inflammatory bioactive lipid concentration. Specifically, Prostaglandins D2, E3, E2, and E3 from the COX pathway, and (±)12,13-DiHOME from the CYP pathway, had the greatest number of negative associations with BDI-2 domain scores. Previous animal studies have reported that Prostaglandins D2 induces allergic inflammation,^55^ while Prostaglandins E2 has been implicated in inflammatory responses.^56–58^ Additionally, Prostaglandins D3 and E3 have been shown to induce the expression of Prostaglandin H synthase 2,^59^ which is an enzyme responsible for the overproduction of pro-inflammatory prostaglandins such as PGE2 at inflammatory sites.^60^ Moreover, elevated levels of (±)12,13-DiHOME from the CYP pathway have been associated with activated inflammation and immune response in several animal models.^61^ In contrast, anti-inflammatory oxylipins from different pathways consistently showed positive associations with BDI-2 scores. These oxylipins included 15-deoxy-Δ12,14-prostaglandin-J2 from the COX pathway,^62,63^ 5(6)-EET and 8(9)-EET from the CYP pathway,^64^ and Leukotriene E4^65^, as well as Resolvin D2^66,67^ from LOX pathway. These results indicate that higher pro-inflammatory lipids were associated with poorer neurodevelopment, while higher anti-inflammatory lipids were associated with better neurodevelopmental status.

Similar patterns emerged in the CBCL/1.5–5 analyses, with pro-inflammatory bioactive lipid associated with higher CBCL/1.5–5 scores (indicating more problematic symptoms), while anti-inflammatory bioactive lipids were related to lower CBCL/1.5–5 scores. For example, a pro-inflammatory parent compounds, LA and AA, and their metabolite oxylipins were positively associated with CBCL/1.5–5 scores. LA is a precursor of AA, which produces pro-inflammatory oxylipins and endocannabinoids. 12(13)-EpOME and (±)12,13-DiHOME – metabolites of LA—and 20-HETE and 20-carboxy arachidonic acid—metabolites of AA—consistently showed positive associations with CBCL/1.5–5 scores. Another metabolite, LOX-derived 13-HODE, has demonstrated both pro- and anti-inflammatory activities in previous studies^68,69^ but showed strong positive associations with CBCL/1.5–5 scores in the present study. In contrast, 5,6-EET from the CYP pathway^70^ and Leukotriene E4^65^ from the LOX pathway, both known for their anti-inflammatory properties, showed negative associations—suggesting that lower levels of these oxylipins may be linked to more problematic symptoms.

These consistent trends suggest that an activated inflammatory response in pregnancy, induced by altered bioactive lipid levels during pregnancy, may serve as a potential mechanism adversely affecting child neurodevelopment and behavioral outcomes. A recent epidemiological study demonstrated that maternal oxylipin levels during pregnancy predict elevated pro-inflammatory cytokine concentrations in both mothers and infants.^71^ Additionally, another study found that oxylipins metabolized from DHA and EPA mediate the anti-inflammatory effects of their parent compounds by attenuating pro-inflammatory cytokines.^72^ Both experimental and epidemiological studies have convergently shown that activated maternal inflammation induced by bioactive lipid during pregnancy negatively impacts fetal brain development.^4,73^ Maternal inflammation during pregnancy may affect the developing brain and central nervous system through various mechanisms, such as altering brain structure, disrupting axonal growth, and generating reactive oxygen species (ROS) in the fetal brain.^74^

Moreover, bioactive lipids may directly influence the development of brain and neuron morphology. For example, within the brain’s microvasculature, Prostaglandin E2 enhances permeability and compromises the integrity of the blood-brain barrier.^75,76^ Recent animal studies have shown that LA and several of its oxylipin metabolites—including 9-HODE, 13-HODE, 9-OxoODE, and 12(13)-EpOME—significantly influence neurodevelopmental processes, with 9-HODE and 13-HODE altering axonal outgrowth, and 9-HODE, 9-OxoODE, and 12(13)-EpOME affecting dendritic arborization.^77,78^ Furthermore, another recent study demonstrated that Prostaglandin E2 regulates membrane excitability and long-term synaptic plasticity in hippocampal perforant path-dentate gyrus synapses.^79^

Although there is limited information about how maternal bioactive lipid during pregnancy differentially affect neurodevelopment and behaviors by child sex, several possible explanations exist. One such explanation involves sex-specific responses to maternal inflammation. Research suggests that male fetuses might be more vulnerable to inflammatory insults due to less robust maternal anti-inflammatory responses compared to mothers carrying female fetuses.^80,81^ Given that the bioactive lipids examined in our study exhibit either pro- or anti-inflammatory properties, the observed sex differences may reflect differential vulnerability to bioactive lipid–driven inflammatory responses. Moreover, oxylipin-related enzymes may interact with sex hormones.^82^ For example, animal studies have shown that estrogen increases the expression of 5-lipoxygenase (5-LO), a key enzyme in the LOX pathway involved in metabolizing AA to leukotrienes or converting 15-HETE to lipoxins.^83^ In contrast, progesterone was found to rapidly down-regulate the biosynthesis of 5-LO.^83^ Sex hormones play vital roles in pregnancy and gestational sex hormone concentrations differ by fetal sex, supporting the idea that their interaction with oxylipin-related enzymes could account for observed dimorphism by fetal sex. Furthermore, Hennebelle et al.^77^ demonstrated that male and female rat pups have different brain oxylipin distributions. For instance, in female pup brains, a higher percentage of EPA- and DHA-derived metabolites accounted for the total oxylipins compared to male pup brains. Given the innate differences in fetal oxylipin distribution by sex, even slight changes in oxylipin levels might generate imbalances in the oxylipin system in different ways for males and females. Indeed, two earlier mentioned animal studies^77^ showed that oxylipins and parent compound affect neuromorphology in a sex-specific manner, with LA increasing axonal outgrowth only in females, while 13-HODE raised axonal outgrowth only in males. Still, the limited information about the sex-specific effects of maternal bioactive lipids on NDP and EBP calls for further research to understand the etiology.

Numerous epidemiological studies have shown that children born preterm are at a higher risk for severe neurodevelopmental disabilities such as low IQ, ASD, ADHD, and even social and psychiatric impairments.^84–87^ This increased susceptibility is likely due to the significant brain development that occurs in the later stages of pregnancy. Specifically, the third trimester of pregnancy is a crucial period for fetal brain plasticity, since rapid cellular events such as synaptogenesis, neuronal migration, and myelination, which are essential for the formation of neural circuits, occur during this stage.^88^ Interestingly, oxylipins play a crucial role in onset of parturition^89,90^ and oxylipin levels during pregnancy have been associated with a higher risk of preterm birth.^91^ Specifically, our previous study with the LIFECODES birth cohort in Boston demonstrated a link between maternal oxylipin and parent compound PUFA levels and adverse pregnancy outcomes.^92^ Our findings in the context of prior research point to a potential association between oxylipin levels during pregnancy and child NDP and EBP, possibly involving mechanisms related to preterm birth. Future research examining the mediating role of preterm birth will further enhance our understanding of the underlying etiology.

A notable strength of our study is the inclusion of comprehensive bioactive lipid data—including oxylipins and their parent PUFAs—spanning multiple metabolic pathways, which allowed us to better explore the underlying biological mechanisms. Additionally, we utilized two validated assessments to examine NDP and EBP across multiple domains and aspects. Furthermore, we observed child-sex-specific differences and differences by preterm birth status in the associations between maternal bioactive lipids and child NDP and EBP, highlighting potentially more vulnerable populations. However, this study has some limitations. Firstly, we only measured maternal bioactive lipid levels at a later stage of gestation. while concentrations may fluctuate throughout pregnancy and different levels in each trimester could differently impact brain development. Although the later stages of gestation are marked by rapid neuronal development and brain growth, the earlier stages are also critical, as foundational brain structures are established during the first trimester.^93^ Therefore, future research that measures bioactive lipid concentrations at multiple time points across pregnancy will enhance our understanding of their relationships with child NDP and EBP. Additionally, we stratified our study sample to explore potential effect modification by child sex, based on evidence from previous studies suggesting sex-specific vulnerability. However, this approach reduced the sample size, and the findings should be interpreted with caution and confirmed in larger studies. Lastly, given the exploratory nature of our analysis and the limited statistical power, we did not adjust for multiple comparisons or conduct further age-specific analyses. Therefore, future studies with larger sample sizes are needed to implement rigorous control for multiple testing and to examine age-specific effects in addition to the potential mediating role of preterm birth or other factors.

## CONCLUSION

Our analyses indicate that maternal bioactive lipids during pregnancy are associated with NDP and EBP in children aged 1–3 years. Notably, distinct trends based on inflammatory activities of bioactive lipids and sex-specific associations were observed. After excluding preterm birth cases, the observed associations were largely attenuated. These findings contribute to the growing understanding of the role of bioactive lipids in fetal brain development and highlight potential pathways that could inform early interventions aimed at reducing the risk of NDP and EBP in early childhood. Still, further research is needed to examine more comprehensive biomolecular mechanisms through which bioactive lipid-related maternal physiological changes impact neurodevelopment and behavior in early childhood.

## Supplementary Material

Supp Figures

Supp Tables

ADDITIONAL INFORMATION

**Supplementary information** The online version contains supplementary material available at https://doi.org/10.1038/s41390-025-04465-4.

## Figures and Tables

**Table 1.** Demographic characteristics of the study population.

	Category	BDI-2 *N* = 143 *N* (%)	CBCL/1.5–5 *N* = 215 *N* (%)
Maternal age (years)	18–24	42 (29.4%)	58 (27%)
	25–29	46 (32.2%)	75 (34.9%)
	30–34	30 (21%)	52 (24.2%)
	35–41	25 (17.5%)	30 (14%)
Maternal education	GED or less	19 (13.3%)	34 (15.8%)
	Some college	50 (35%)	76 (35.3%)
	Bachelors or higher	73 (51%)	103 (47.9%)
	Missing	1 (0.7%)	2 (0.9%)
Currently employed	No	49 (34.3%)	80 (37.2%)
	Yes	93 (65%)	134 (62.3%)
	Missing	1 (0.7%)	1 (0.5%)
Pre-pregnancy BMI (kg/m^2^)	≤25	70 (49%)	97 (45.1%)
	>25–<30	38 (26.6%)	61 (28.4%)
	≥30	26 (18.2%)	48 (22.3%)
	Missing	9 (6.3%)	9 (4.2%)
Marital status	Single	18 (12.6%)	25 (11.6%)
	Married	82 (57.3%)	112 (52.1%)
	Cohabitating	40 (28%)	75 (34.9%)
	Missing	3 (2.1%)	3 (1.4%)
Annual household income	<10k	38 (26.6%)	68 (31.6%)
	10k–<30k	35 (24.5%)	48 (22.3%)
	30k–<50k	36 (25.2%)	50 (23.3%)
	≥50k	18 (12.6%)	18 (8.4%)
	Missing	16 (11.2%)	31 (14.4%)
Smoking status	Never	132 (92.3%)	191 (88.8%)
	Ever	9 (6.3%)	22 (10.2%)
	Current	2 (1.4%)	2 (0.9%)
Alcohol use	Never	76 (53.1%)	130 (60.5%)
	Yes, before pregnancy	62 (43.4%)	78 (36.3%)
	Yes, currently	4 (2.8%)	6 (2.8%)
	Missing	1 (0.7%)	1 (0.5%)
Number of pregnancy	0	59 (41.3%)	92 (42.8%)
	1	50 (35%)	67 (31.2%)
	2–5	34 (23.8%)	56 (26%)
Birth outcome	Term birth (≥37 weeks)	130 (90.9%)	199 (92.6%)
	Preterm birth (<37 weeks)	13 (9.1%)	16 (7.4%)
Child sex	Female	77 (53.8%)	110 (51.2%)
	Male	66 (46.2%)	105 (48.8%)
Child age (months)	mean ± SD	22.4 ± 9.7	24.4 ± 7.1

**Table 2.** Distributions of bioactive lipid concentrations in the study population.

Group name	Bioactive lipids	GM (IQR)	
		BDI-2 sample (*N* = 143)	CBCL/1.5–5 sample (*N* = 215)
Cyclooxygenase	13,14-dihydro-15-keto prostaglandin D2	7.2 (2.58,16.69)	3.79 (1.89,6.97)
	13,14-dihydro-15-keto prostaglandin E2	5.35 (2.44,9.88)	3.68 (1.93,8.34)
	13,14-dihydro-15-keto prostaglandin F2α	15.35 (8.52,29.62)	12.96 (8.06,22.98)
	13,14-dihydro-15-keto prostaglandin J2	2.81 (1.22,8.71)	3.61 (1.83,8.52)
	15-deoxy-Δ12,14-prostaglandin J2	2.89 (1.18,6.84)	2.33 (0.76,5.8)
	9-OxoODE	142.38 (134.77,197.69)	148.33 (134.06,200.76)
	Bicyclo prostaglandin E1	1.38 (0.79,2.34)	1.53 (0.86,2.61)
	Bicyclo prostaglandin E2	0.65 (0.32,1.09)	0.87 (0.32,1.36)
	Prostaglandin A2	16.48 (15.54,20.87)	14.24 (13.2,20.01)
	Prostaglandin B2	8.77 (4.13,22.83)	7.38 (2.34,21.91)
	Prostaglandin D2	10.16 (4.1,22.74)	7.94 (3.02,17.68)
	Prostaglandin D3	4.69 (2.04,8.81)	3.8 (1.69,7.8)
	Prostaglandin E1 (power)	5.11 (2.5,11.7)	7.71 (3.16,18.85)
	Prostaglandin E2	2.25 (1.17,3.46)	2.16 (1.14,3.46)
	Prostaglandin E3	1.14 (0.51,2.1)	1.16 (0.56,2.16)
	Prostaglandin J2	12.7 (8.77,19.62)	12.08 (8.38,19.39)
	Thromboxane B2	3.55 (1.57,8.96)	3.4 (1.59,7.61)
Cytochrome p450	(±)11,12-DHET	1.08 (0.7,1.29)	1.17 (0.7,1.56)
	(±)12,13-DiHOME	16.6 (10.28,27.35)	18.85 (10.59,32.71)
	(±)18-HETE	2.87 (1.96,6.19)	3.45 (2.57,6.58)
	(±)5,6-DHET	1.53 (0.8,3.69)	1.88 (0.75,4.45)
	(±)8,9-DHET	2.11 (0.88,3.98)	2.51 (1,5.19)
	(±)9,10-DiHOME	12.64 (6.6,18.56)	14.94 (7.88,28.09)
	11(12)-EET	2.97 (1.35,5.93)	2.49 (1.17,4.49)
	11(S)-HETE	1.37 (0.58,3.32)	1.87 (0.74,4.83)
	12(13)-EpOME	16.24 (11.88,32.12)	15.36 (12.16,30.3)
	14(15)-EET	3.96 (2.09,8.07)	4.27 (2.28,8.63)
	16(S)-HETE	3.3 (1.14,10.44)	4.82 (1.75,12.73)
	17(S)-HETE	3.33 (2.04,8.44)	4.32 (3.43,8.11)
	20(S)-HETE	138.71 (113.24,263.25)	156.81 (132.45,270.23)
	20-carboxy arachidonic acid	18.42 (11.32,91.94)	34.95 (18.51,105.54)
	5(6)-EET	36.13 (12.07,194.23)	25.95 (6.79,104.33)
	8(9)-EET	8.68 (3.98,20.12)	6.15 (2.41,15.38)
	9(10)-EpOME	9.81 (7.03,16.76)	10.09 (7.7,16.27)
	9s-HODE	14.19 (8.66,25.54)	15.61 (9.59,27.13)
Lipoxygenase	12(S)-HETE	3.03 (1.47,4.67)	2.61 (1.34,4.51)
	12-OxoETE	7.34 (3.26,17.15)	8.14 (3.54,17.2)
	13-OxoODE	23.51 (17.52,35.99)	32.12 (21.52,41.26)
	13S-HODE	23 (14.09,32.03)	27.62 (17.26,42.66)
	15(S)-HETE	1.85 (0.9,3.38)	2.07 (0.99,3.61)
	15-OxoETE	15.76 (5.16,43.84)	13 (3.75,41.82)
	5(S)-HETE	5.4 (2.31,11.79)	6 (2.33,12.45)
	5-OxoETE	21.29 (7.77,64.24)	20.21 (8.18,42.81)
	8(S)-HETE	1.39 (0.65,3.14)	1.44 (0.6,3.31)
	Leukotriene B4	1.01 (0.44,2.04)	0.57 (0.28,1.19)
	Leukotriene D4	6.64 (2.62,19.3)	8 (2.88,22.94)
	Leukotriene E4	0.28 (0.07,1.29)	NA (0.05,0.74)
	Resolvin D1	11.33 (7.17,22.7)	12.65 (7.31,23.64)
	Resolvin D2	8 (4.12,13.38)	8.91 (5.66,14.06)
Parent compound PUFAs	Arachidonic acid	13.08 (7.7,20.08)	11.1 (7.2,18.44)
	Docosahexaenoic acid	5.12 (4.11,11.13)	7.03 (5.04,11.62)
	Eicosapentaenoic acid	2.05 (1.06,3.12)	2.06 (1.09,3.05)
	Linoleic acid	67.87 (40.19,144.16)	100.51 (59.39,184.48)
	α-Linolenic acid	341.59 (275.91,988.51)	562.77 (399.94,1296.47)

*GM* geometric mean, *IQR* interquartile range. Units are μmol/L for docosahexaenoic acid (DHA), eicosapentaenoic acid (EPA), linoleic acid (LA), and α-linolenic acid (ALA), and nmol/L, otherwise.

**Table 3.** Distribution of neurodevelopmental (BDI-2) and behavioral (CBCL/1.5–5) assessment scores in the study population by child sex and possible range.

BDI	All				Male				Female				Possible range
	Q25	Q50	Q75	Range	Q25	Q50	Q75	Range	Q25	Q50	Q75	Range	
Adaptive	95	103	108	[55,125]	95	100	105	[55,125]	99	105	110	[75,120]	[55, 145]
Cognitive	90	97	103	[63,117]	87	99	103	[65,117]	90	96	103	[63,117]	
Communication	92	100	108	[55,138]	88	100	105	[55,138]	93	100	108	[62,127]	
Motor	98	104	110	[72,125]	95	103	108	[72,120]	100	104	110	[83,125]	
Personal-social	102	105	110	[60,133]	100	105	110	[60,120]	103	105	113	[73,133]	
Total	98	104	108	[68,126]	97	104	107	[73,118]	98	104	109	[68,126]	
CBCL	Q25	Q50	Q75	Range	Q25	Q50	Q75	Range	Q25	Q50	Q75	Range	Possible range
Externalizing score	5	9	14	[0,37]	5	9	14	[0,27]	5	9	13	[0,37]	[0, 48]
Internalizing score	1	3	7	[0,27]	1	3	6	[0,27]	1	3	7	[0,26]	[0, 72]
Total score	12	21	31	[0,100]	12	21	31	[0,83]	12	22	31	[0,100]	[0, 184]

## Data Availability

The data used to support the findings of this study are available from the corresponding author upon reasonable request.

## IMPACT:

- Previous studies suggest potential roles of maternal PUFAs and oxylipins during pregnancy on child NDP and EBP, as these bioactive lipids play crucial roles in immune response, inflammation, and oxidative stress.
- In this cohort study, we found several associations between maternal bioactive lipid levels during pregnancy and child neurodevelopment and behavior from 1 to 3 years of age.
- The associations were attenuated after excluding preterm children, suggesting a potential mediating role of preterm birth. Additionally, distinct patterns emerged based on both the inflammatory properties of the bioactive lipids and child sex.
