# Supplementary material for: Maternal bioactive lipids during pregnancy and early childhood neurodevelopment and behavior
Source: Pediatr Res. 2025 Oct 8;99(5):1880–91. doi: 10.1038/s41390-025-04465-4 (PMC12914742; doi:10.1038/s41390-025-04465-4)
Supplement: Supplementary file 1 — Supplementary Figures [file 41390_2025_4465_MOESM1_ESM.pdf]

Supplementary Figures

Maternal Bioactive Lipids during Pregnancy and Early Childhood Neurodevelopment and Behavior

Seonyoung Park [a], Megan Woodbury [b], Sung Kyun Park [a,c], Bhramar Mukherjee [d], Wei Hao [d], Lixia Zeng [e], Subramaniam Pennathur [e,f], Gredia Huerta Montañez [g], Zaira Rosario Pabón [g], Carmen M. Vélez Vega [h], José F. Cordero [i], Akram Alshawabkeh [b], Deborah J. Watkins [a], John D. Meeker [a*]

[a] Department of Environmental Health Sciences, University of Michigan School of Public Health, Ann Arbor, MI 48109, USA
[b] Department of Civil and Environmental Engineering, Northeastern University, Boston, MA 02115, USA
[c] Department of Epidemiology, University of Michigan School of Public Health, Ann Arbor, MI 48109, USA
[d] Department of Biostatistics, Yale University School of Public Health, New Haven, CT 06510, USA
[e] Department of Biostatistics, University of Michigan School of Public Health, Ann Arbor, MI 48109, USA
[f] Department of Internal Medicine, University of Michigan, Ann Arbor, MI 48109, USA
[g] Department of Molecular and Integrative Physiology, University of Michigan, Ann Arbor, MI 48109, USA
[h] Department of Electrical and Computer Engineering, Northeastern University, Boston, MA 02115, USA
[i] Department of Social Sciences, UPR Medical Sciences Campus, University of Puerto Rico Graduate School of Public Health, San Juan, PR 00936-5067, USA
[j] Department of Epidemiology and Biostatistics, University of Georgia, Athens, GA 30606, USA

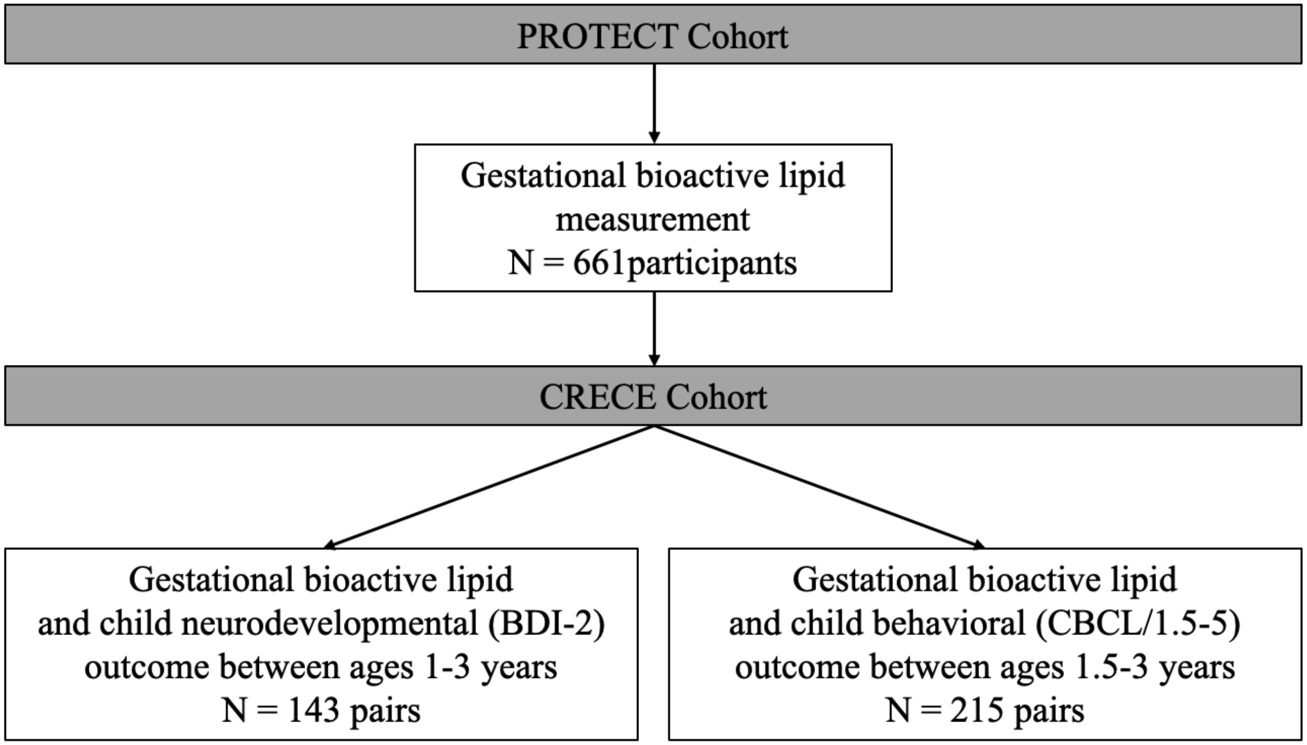

Supplementary Figure S1. Flowchart illustrating analysis sample sizes. This study includes 259 mother-child pairs with gestational bioactive lipid measurement and childhood neurodevelopmental (143 pairs) or behavioral outcome (215 pairs) measurement.

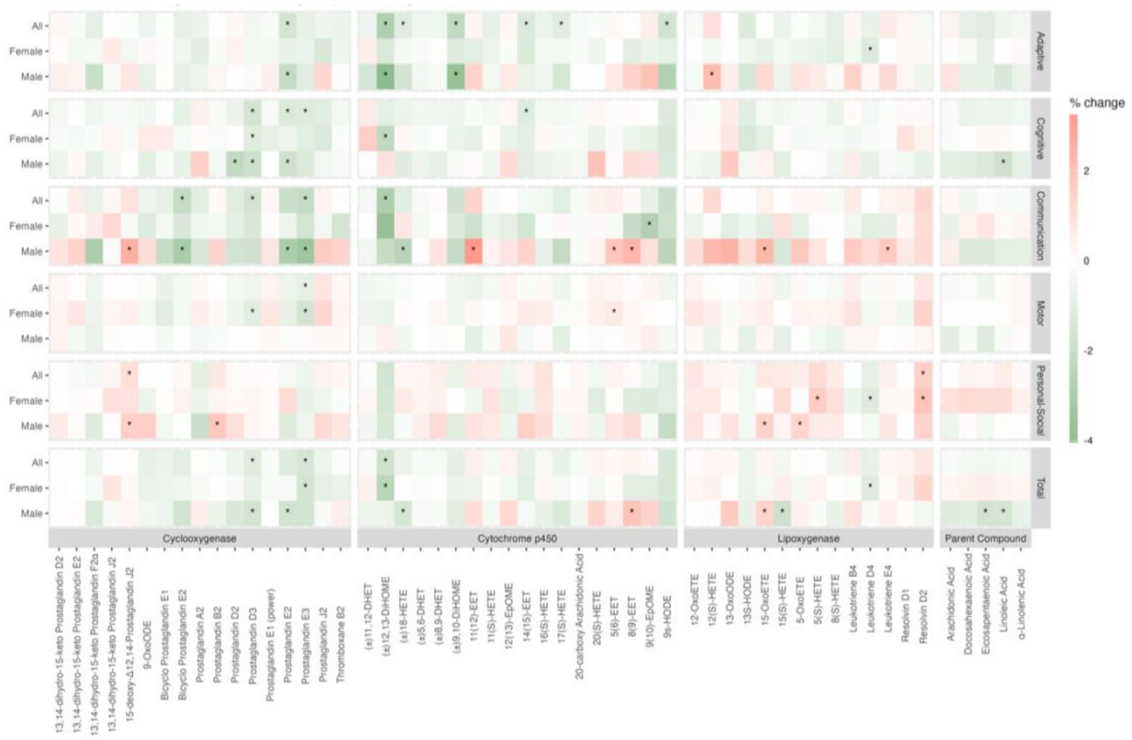

Supplementary Figure S2. Percentage change in BDI-2 for a doubling increase in maternal bioactive lipid concentrations by child sex without adjusting for dietary fatty acid consumption. The number of participants and sample size for each group were as follows: N_{all}=143 (192), N_{Female}=77 (110), N_{Male}=66 (82). Asterisk (*) indicates statistical significance ($p < 0.05$).

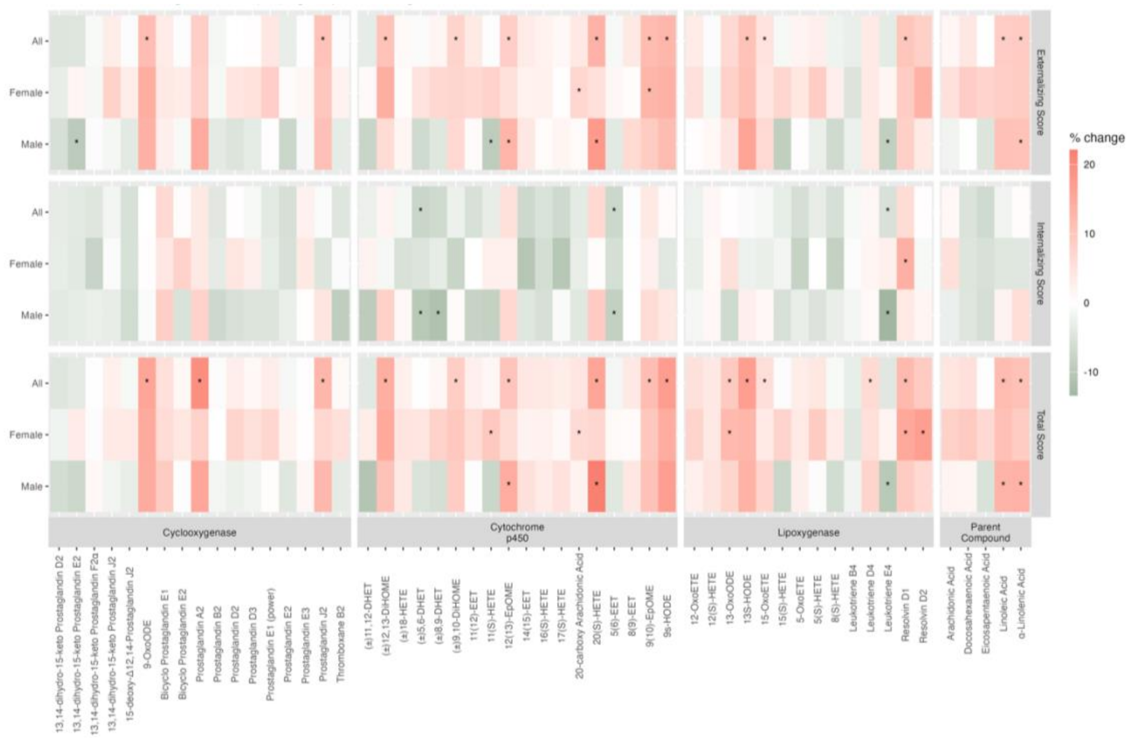

Supplementary Figure S3. Percent change in CBCL/1.5-5 for a doubling increase in maternal bioactive lipid concentrations by child sex without adjusting for dietary fatty acid consumption. The number of participants and sample size for each group were as follows: N_{all}=215 (293), N_{Female}=110 (151), N_{Male}=105 (142). Asterisk (*) indicates statistical significance ($p < 0.05$).

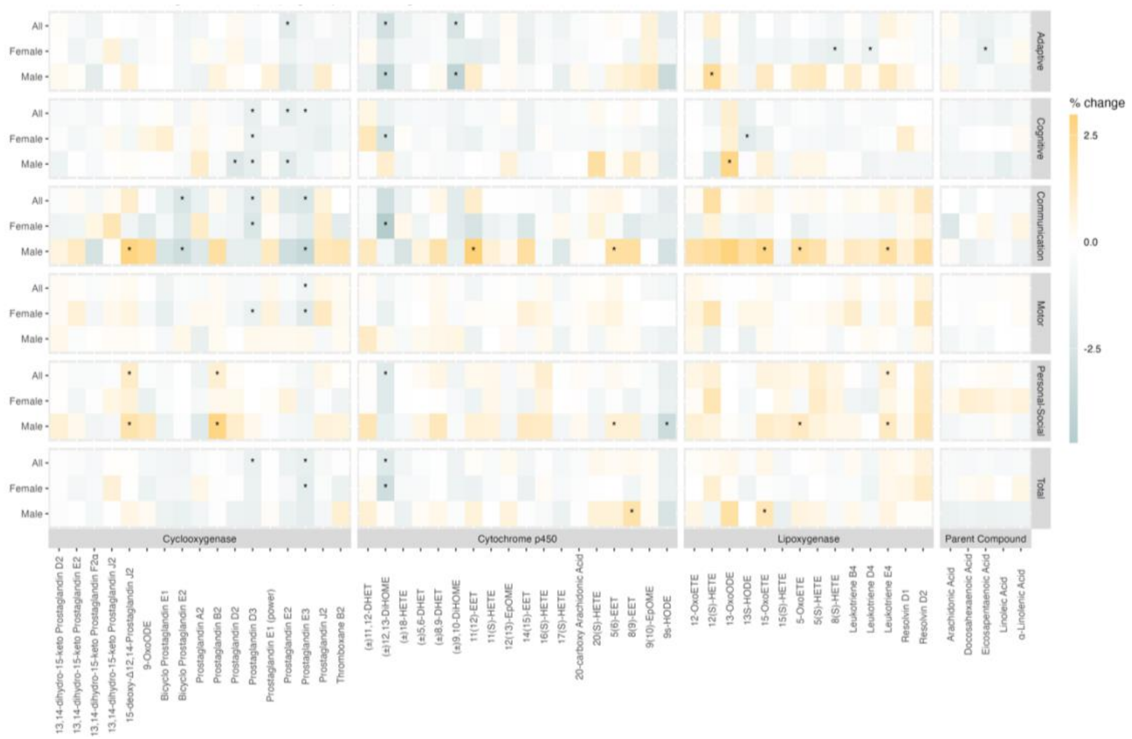

Supplementary Figure S4. Percentage change in BDI-2 for a doubling increase in maternal bioactive lipid concentrations over gestation among term birth children by child sex among term birth children. The number of participants and sample size for each group were as follows: N_{all}=130 (176), N_{Female}=69 (99), N_{Male}=61 (77). Asterisk (*) indicates statistical significance ($p < 0.05$).

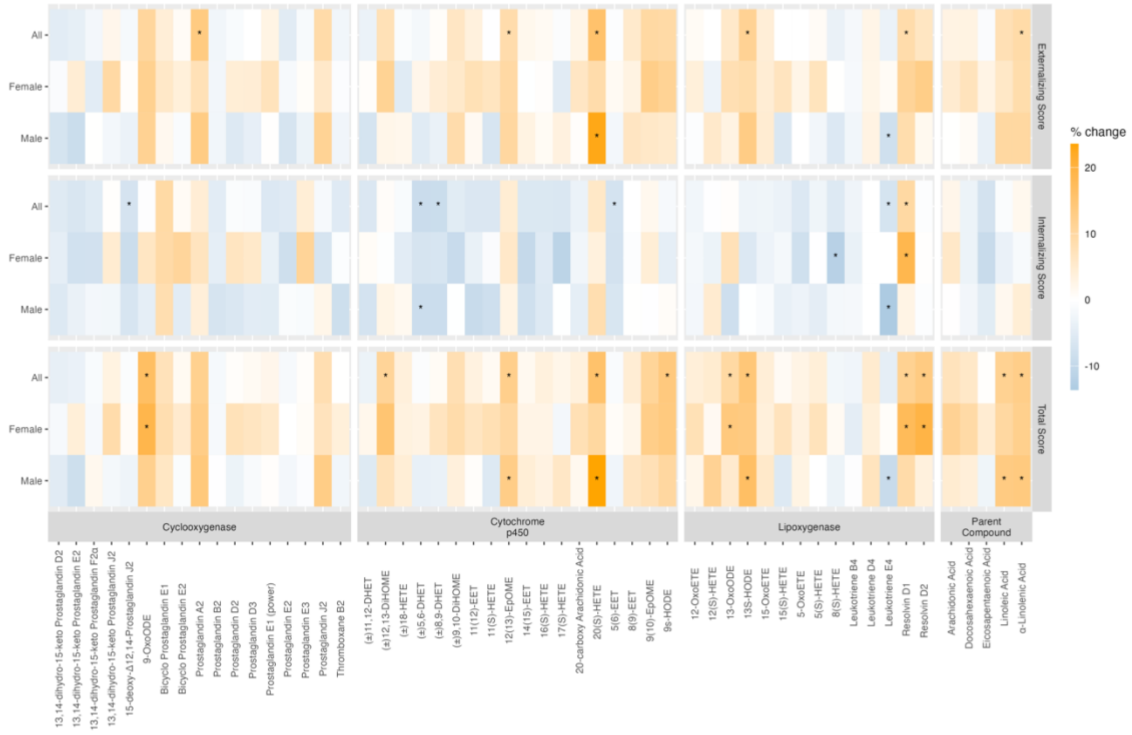

Supplementary Figure S5. Percent change in CBCL/1.5-5 for a doubling increase in maternal bioactive lipid concentrations by child sex among term birth children. The number of participants and sample size for each group were as follows: N_{all}=199 (271), N_{Female}=98 (133), N_{Male}=101 (138). Asterisk (*) indicates statistical significance ($p < 0.05$).
