# Supplementary material for: Maternal bioactive lipids during pregnancy and early childhood neurodevelopment and behavior
Source: Pediatr Res. 2025 Oct 8;99(5):1880–91. doi: 10.1038/s41390-025-04465-4 (PMC12914742; doi:10.1038/s41390-025-04465-4)
Supplement: Supplementary file 2 — Supplementary Tables [file 41390_2025_4465_MOESM2_ESM.pdf]

Supplementary Tables

Maternal Bioactive Lipids during Pregnancy and Early Childhood Neurodevelopment and Behavior

Seonyoung Park [a], Megan Woodbury [b], Sung Kyun Park [a,c], Bhramar Mukherjee [d], Wei Hao [d], Lixia Zeng [e], Subramaniam Pennathur [e,f], Gredia Huerta Montañez [g], Zaira Rosario Pabón [g], Carmen M. Vélez Vega [h], José F. Cordero [i], Akram Alshawabkeh [b], Deborah J. Watkins [a], John D. Meeker [a*]

[a] Department of Environmental Health Sciences, University of Michigan School of Public Health, Ann Arbor, MI 48109, USA
[b] Department of Civil and Environmental Engineering, Northeastern University, Boston, MA 02115, USA
[c] Department of Epidemiology, University of Michigan School of Public Health, Ann Arbor, MI 48109, USA
[d] Department of Biostatistics, Yale University School of Public Health, New Haven, CT 06510, USA
[e] Department of Biostatistics, University of Michigan School of Public Health, Ann Arbor, MI 48109, USA
[f] Department of Internal Medicine, University of Michigan, Ann Arbor, MI 48109, USA
[g] Department of Molecular and Integrative Physiology, University of Michigan, Ann Arbor, MI 48109, USA
[h] Department of Electrical and Computer Engineering, Northeastern University, Boston, MA 02115, USA
[i] Department of Social Sciences, UPR Medical Sciences Campus, University of Puerto Rico Graduate School of Public Health, San Juan, PR 00936-5067, USA
[j] Department of Epidemiology and Biostatistics, University of Georgia, Athens, GA 30606, USA

Supplementary Table S1. BDI-2 DQ scores by participant characteristics of the 143 mother-child pairs (192 observations).

covariate	level	Adaptive	Personal-Social	Communication	Cognitive	Motor	Total
Maternal Age (years)	18-24	105 [100, 110]	105 [102, 113]	100 [95, 105]	97 [89, 103]	103 [98, 110]	104 [97, 109]
	25-29	100 [95, 110]	106 [104, 113]	103 [89, 108]	97 [90, 103]	107 [102, 110]	104 [98, 108]
	30-34	105 [96, 105]	108 [103, 112]	98 [90, 105]	100 [89, 103]	100 [96, 109]	104 [98, 106]
	35-41	100 [90, 105]	103 [100, 106]	98 [73, 110]	96 [90, 103]	102 [97, 109]	101 [98, 106]
	p-value	*0.18*	*0.08*	*0.76*	*0.98*	***0.02***	*0.74*
Maternal Education	GED or less	105 [100, 110]	105 [100, 114]	100 [94, 107]	93 [90, 103]	104 [100, 112]	104 [96, 110]
	Some college	105 [95, 110]	105 [102, 110]	100 [93, 106]	97 [91, 103]	102 [98, 108]	104 [99, 107]
	Bachelors or higher	100 [95, 106]	107 [103, 111]	100 [88, 108]	100 [89, 103]	105 [100, 110]	104 [98, 108]
	p-value	*0.37*	*0.48*	*0.93*	*0.87*	*0.42*	*0.87*
Currently Employed	No	103 [95, 110]	105 [100, 108]	99 [93, 105]	97 [89, 103]	103 [100, 108]	103 [96, 106]
	Yes	102 [95, 108]	107 [103, 113]	103 [90, 108]	97 [90, 103]	104 [98, 110]	104 [99, 109]
	p-value	*0.82*	***0.04***	*0.48*	*0.79*	*0.71*	*0.33*
Pre-pregnancy BMI (kg/m^2)	(0, 25]	100 [95, 108]	105 [102, 112]	97 [92, 105]	93 [90, 103]	103 [98, 110]	102 [98, 106]
	(25, 29.9]	105 [95, 110]	107 [103, 110]	105 [94, 109]	95 [88, 103]	104 [100, 109]	104 [101, 109]
	(29.9, 51]	100 [95, 105]	103 [99, 108]	104 [87, 109]	100 [92, 103]	105 [97, 112]	99 [95, 109]
	p-value	*0.89*	*0.18*	*0.19*	*0.51*	*0.71*	*0.51*
Marital Status	Single	105 [100, 110]	107 [105, 110]	100 [92, 105]	96 [84, 103]	102 [98, 106]	101 [96, 106]
	Married	100 [95, 108]	105 [102, 113]	103 [92, 108]	97 [90, 103]	104 [98, 110]	104 [99, 109]
	Cohabitating	102 [95, 105]	105 [98, 108]	98 [93, 105]	95 [91, 103]	103 [100, 109]	101 [96, 106]
	p-value	*0.24*	*0.3*	*0.39*	*0.88*	*0.61*	*0.36*
Annual Household Income	<10k	105 [100, 110]	105 [102, 108]	100 [94, 107]	93 [87, 103]	103 [100, 108]	102 [96, 108]
	10k - <30k	100 [95, 105]	105 [102, 113]	100 [95, 107]	97 [90, 103]	104 [98, 109]	104 [99, 108]
	30k - <50k	100 [95, 108]	108 [102, 112]	103 [91, 108]	100 [93, 103]	105 [98, 110]	104 [98, 109]
	≥50k	105 [95, 110]	110 [105, 115]	100 [84, 110]	93 [89, 103]	104 [100, 112]	103 [96, 111]
	p-value	*0.72*	*0.39*	*0.99*	*0.9*	*0.98*	*0.94*
Smoking Status	Never	103 [95, 108]	105 [103, 110]	100 [92, 108]	97 [89, 103]	104 [98, 110]	104 [98, 108]
	Ever	105 [101, 109]	104 [92, 115]	103 [100, 105]	94 [92, 98]	103 [100, 107]	103 [103, 110]
	Current	95 [92, 98]	100 [98, 102]	92 [90, 94]	102 [101, 102]	104 [101, 107]	98 [98, 99]
	p-value	*0.44*	*0.47*	*0.45*	*0.52*	*0.99*	*0.48*
Alcohol Use	Never	104 [95, 108]	105 [100, 110]	100 [93, 108]	97 [90, 103]	104 [98, 110]	102 [97, 106]
	Yes, before pregnancy	100 [95, 105]	105 [103, 112]	100 [90, 108]	94 [88, 103]	104 [100, 109]	104 [98, 109]
	Yes, currently	105 [104, 108]	113 [106, 118]	104 [98, 108]	96 [94, 99]	102 [100, 107]	106 [101, 109]
	p-value	*0.56*	*0.16*	*0.81*	*0.76*	*0.87*	*0.72*
Number of Pregnancy	0	105 [95, 110]	107 [103, 113]	100 [92, 108]	100 [89, 103]	104 [98, 110]	104 [98, 109]
	1	102 [96, 108]	105 [100, 111]	103 [93, 108]	97 [90, 100]	105 [101, 110]	104 [98, 108]
	2 - 5	100 [90, 105]	105 [100, 110]	97 [90, 106]	93 [89, 103]	102 [98, 108]	101 [98, 106]
	p-value	*0.32*	*0.16*	*0.72*	*0.43*	*0.14*	*0.65*
Birth Outcome	Term birth	103 [95, 108]	105 [103, 112]	100 [92, 108]	97 [89, 103]	104 [98, 110]	104 [98, 108]
	Preterm birth	105 [98, 108]	105 [98, 109]	100 [90, 111]	103 [92, 105]	102 [98, 111]	99 [96, 112]
	p-value	0.59	0.49	0.77	0.18	0.9	0.89
Child Sex	Female	105 [99, 110]	105 [103, 113]	100 [93, 108]	96 [90, 103]	104 [100, 110]	104 [98, 109]
	Male	100 [95, 105]	105 [100, 110]	100 [88, 105]	99 [87, 103]	103 [95, 108]	104 [97, 107]
	p-value	*0.18*	*0.14*	*0.07*	*0.82*	***0.04***	*0.5*

Supplementary Table S2. CBCL/1.5-5 composite scores by participant characteristics of the 215 mother-child pairs (293 observations).

covariate	level	Internalizing Score	Externalizing Score	Total Score
Maternal Age (years)	18-24	4 [2, 8]	9 [5, 15]	24 [12, 37]
	25-29	3 [1, 5]	9 [5, 13]	20 [11, 27]
	30-34	3 [1, 7]	10 [6, 13]	23 [12, 33]
	35-41	3 [1, 6]	8 [5, 12]	22 [13, 30]
	p-value	*0.11*	*0.85*	*0.31*
Maternal Education	GED or less	3 [2, 5]	9 [6, 13]	20 [15, 30]
	Some college	4 [1, 8]	8 [3, 14]	23 [10, 36]
	Bachelors or higher	3 [1, 6]	9 [6, 14]	21 [13, 30]
	p-value	*0.28*	*0.8*	*0.87*
Currently Employed	No	3 [1, 7]	8 [4, 13]	21 [12, 33]
	Yes	3 [1, 6]	10 [5, 14]	22 [13, 31]
	p-value	*0.53*	*0.23*	*0.45*
Pre-pregnancy BMI (kg/m^2)	(0, 25]	3 [1, 5]	9 [5, 13]	21 [11, 30]
	(25, 29.9]	4 [2, 6]	9 [5, 13]	20 [13, 30]
	(29.9, 51]	5 [1, 8]	10 [5, 14]	24 [16, 39]
	p-value	***0.02***	*0.75*	*0.24*
Marital Status	Single	4 [2, 7]	11 [7, 17]	25 [21, 40]
	Married	3 [1, 6]	8 [5, 13]	20 [12, 30]
	Cohabitating	3 [2, 7]	8 [4, 13]	22 [12, 31]
	p-value	*0.48*	*0.06*	*0.12*
Annual Household Income	<10k	3 [1, 8]	9 [5, 15]	23 [12, 38]
	10k - <30k	3 [1, 5]	8 [3, 12]	20 [11, 27]
	30k - <50k	4 [2, 6]	10 [6, 15]	23 [16, 35]
	≥50k	3 [1, 6]	9 [5, 12]	19 [13, 29]
	p-value	*0.73*	*0.16*	*0.16*
Smoking Status	Never	3 [1, 6]	9 [5, 13]	21 [12, 30]
	Ever	4 [2, 9]	11 [4, 16]	28 [9, 40]
	Current	16 [15, 16]	22 [21, 23]	64 [62, 67]
	p-value	***0.04***	*0.06*	***0.03***
Alcohol Use	Never	3 [1, 6]	8 [4, 13]	19 [12, 29]
	Yes, before pregnancy	4 [1, 8]	10 [6, 15]	24 [14, 35]
	Yes, currently	6 [2, 8]	10 [6, 16]	31 [13, 39]
	p-value	*0.19*	*0.19*	*0.1*
Number of Pregnancy	0	4 [1, 7]	10 [5, 14]	22 [13, 35]
	1	3 [1, 6]	8 [5, 12]	20 [11, 30]
	2 - 5	3 [1, 6]	8 [4, 12]	20 [14, 31]
	p-value	*0.48*	*0.19*	*0.38*
Birth Outcome	Term birth	3 [1, 6]	9 [5, 13]	21 [12, 31]
	Preterm birth	6 [3, 11]	10 [4, 16]	26 [15, 45]
	p-value	***0.04***	*0.62*	*0.17*
Child Sex	Female	3 [1, 7]	9 [5, 13]	22 [12, 31]
	Male	3 [1, 6]	9 [5, 14]	21 [12, 31]
	p-value	*0.68*	*0.7*	*0.51*

Supplementary Table S3. Percent change of BDI-2 scores by doubling increase in maternal bioactive lipid concentration from the entire study population and stratified by child sex.

**All**							
Group Name	Bioactive Lipids	Adaptive	Cognitive	Communication	Motor	Personal-Social	Total
Cyclooxygenase	Bicyclo Prostaglandin E1	-0.04 (-1.45, 1.37)	0.12 (-1.13, 1.36)	-1.13 (-3.36, 1.1)	-1.24 (-2.3, -0.18)	-0.35 (-1.73, 1.03)	-0.86 (-2.22, 0.51)
Cyclooxygenase	Bicyclo Prostaglandin E2	-0.87 (-2.11, 0.38)	-0.74 (-1.83, 0.35)	-2.2 (-4.12, -0.28)	-0.62 (-1.49, 0.24)	0.11 (-1.14, 1.35)	-1.02 (-2.24, 0.2)
Cyclooxygenase	15-deoxy-Δ12,14-Prostaglandin J2	-0.17 (-1.16, 0.82)	-0.43 (-1.31, 0.44)	0.97 (-0.59, 2.52)	0.11 (-0.67, 0.89)	1.18 (0.23, 2.12)	-0.01 (-0.98, 0.96)
Cyclooxygenase	13,14-dihydro-15-keto Prostaglandin D2	0.23 (-0.74, 1.21)	-0.46 (-1.33, 0.41)	-0.12 (-1.64, 1.4)	0.44 (-0.32, 1.19)	0.09 (-0.84, 1.02)	-0.09 (-1.04, 0.87)
Cyclooxygenase	13,14-dihydro-15-keto Prostaglandin E2	-0.32 (-1.6, 0.97)	-0.24 (-1.33, 0.86)	0.7 (-1.38, 2.79)	0.29 (-0.72, 1.3)	-0.04 (-1.31, 1.22)	0.02 (-1.21, 1.25)
Cyclooxygenase	13,14-dihydro-15-keto Prostaglandin F2α	-0.93 (-2.21, 0.34)	-0.63 (-1.75, 0.5)	-0.67 (-2.7, 1.36)	-0.43 (-1.49, 0.63)	-0.24 (-1.49, 1.02)	-0.59 (-1.83, 0.66)
Cyclooxygenase	13,14-dihydro-15-keto Prostaglandin J2	0 (-0.91, 0.92)	-0.23 (-1.01, 0.56)	0.22 (-1.18, 1.62)	-0.26 (-0.93, 0.41)	0.09 (-0.78, 0.97)	-0.05 (-0.9, 0.8)
Cyclooxygenase	Prostaglandin A2	-0.62 (-2.67, 1.43)	0.5 (-1.29, 2.28)	-1.03 (-4.17, 2.1)	0.2 (-1.48, 1.88)	-1.03 (-2.95, 0.89)	0.01 (-2.1, 2.12)
Cyclooxygenase	Prostaglandin B2	-0.06 (-1, 0.87)	-0.35 (-1.15, 0.44)	0.26 (-1.22, 1.74)	0.16 (-0.58, 0.91)	0.92 (0.01, 1.82)	-0.13 (-1.04, 0.78)
Cyclooxygenase	Prostaglandin D2	0.26 (-0.76, 1.28)	-0.62 (-1.53, 0.29)	-1.16 (-2.71, 0.4)	-0.54 (-1.29, 0.2)	0.04 (-0.93, 1.02)	-0.65 (-1.63, 0.34)
Cyclooxygenase	Prostaglandin D3	-0.47 (-1.45, 0.51)	-1.52 (-2.36, -0.67)	-1.63 (-3.08, -0.18)	-0.65 (-1.34, 0.04)	0.09 (-0.82, 1)	-1.22 (-2.14, -0.3)
Cyclooxygenase	Prostaglandin E1 (power)	-0.25 (-1.19, 0.68)	-0.27 (-1.11, 0.56)	0.23 (-1.21, 1.68)	0.21 (-0.55, 0.97)	-0.05 (-0.96, 0.86)	-0.3 (-1.24, 0.65)
Cyclooxygenase	Prostaglandin E2	-1.7 (-2.98, -0.43)	-1.48 (-2.59, -0.37)	-1.95 (-3.96, 0.06)	-0.48 (-1.44, 0.49)	-0.14 (-1.41, 1.13)	-1.14 (-2.36, 0.08)
Cyclooxygenase	Prostaglandin E3	-0.86 (-2.01, 0.29)	-1.14 (-2.09, -0.19)	-2.26 (-3.99, -0.54)	-1.13 (-1.94, -0.32)	-0.71 (-1.78, 0.36)	-1.59 (-2.59, -0.59)
Cyclooxygenase	Prostaglandin J2	-0.53 (-2.01, 0.95)	-1 (-2.29, 0.3)	0.29 (-2.05, 2.63)	0.61 (-0.55, 1.77)	0.23 (-1.2, 1.66)	-0.74 (-2.18, 0.7)
Cyclooxygenase	Thromboxane B2	-0.64 (-1.62, 0.35)	-0.41 (-1.3, 0.48)	-0.13 (-1.66, 1.4)	0.13 (-0.59, 0.84)	0.26 (-0.7, 1.22)	-0.17 (-1.13, 0.8)
Cyclooxygenase	9-OxoODE	-0.6 (-2.31, 1.11)	-0.37 (-1.85, 1.1)	-0.74 (-3.35, 1.87)	-0.61 (-1.89, 0.67)	-0.22 (-1.85, 1.41)	-1.04 (-2.6, 0.52)
Cytochrome p450	11(S)-HETE	-0.86 (-1.8, 0.08)	-0.43 (-1.25, 0.4)	-0.24 (-1.71, 1.23)	-0.35 (-1.08, 0.38)	0.6 (-0.31, 1.51)	-0.37 (-1.27, 0.54)
Cytochrome p450	(±)11,12-DHET	-0.76 (-2.39, 0.88)	0.56 (-0.85, 1.96)	-0.36 (-2.88, 2.16)	-0.19 (-1.45, 1.07)	0.23 (-1.32, 1.79)	-0.16 (-1.64, 1.32)
Cytochrome p450	11(12)-EET	0.31 (-0.94, 1.56)	-0.22 (-1.28, 0.85)	1.42 (-0.5, 3.34)	-0.57 (-1.53, 0.38)	0.65 (-0.57, 1.86)	0.5 (-0.71, 1.72)
Cytochrome p450	12(13)-EpOME	0.29 (-0.74, 1.32)	0.29 (-0.62, 1.2)	0.01 (-1.56, 1.59)	0.39 (-0.41, 1.19)	-0.64 (-1.6, 0.33)	0.39 (-0.55, 1.33)
Cytochrome p450	(±)12,13-DiHOME	-2.97 (-4.75, -1.2)	-0.82 (-2.44, 0.79)	-3.19 (-6.01, -0.36)	-0.82 (-2.28, 0.65)	-1.89 (-3.64, -0.13)	-1.96 (-3.67, -0.25)
Cytochrome p450	14(15)-EET	-1.42 (-2.68, -0.15)	-1.13 (-2.21, -0.05)	-0.52 (-2.54, 1.5)	-0.67 (-1.68, 0.35)	0.15 (-1.08, 1.39)	-1.07 (-2.24, 0.11)
Cytochrome p450	16(S)-HETE	-0.64 (-1.66, 0.37)	-0.31 (-1.18, 0.55)	-0.52 (-2.08, 1.04)	0.06 (-0.71, 0.84)	0.86 (-0.1, 1.83)	-0.35 (-1.3, 0.59)
Cytochrome p450	17(S)-HETE	-1.12 (-2.17, -0.06)	-0.36 (-1.31, 0.59)	-1.14 (-2.76, 0.48)	-0.84 (-1.62, -0.06)	-0.16 (-1.19, 0.87)	-0.87 (-1.9, 0.16)
Cytochrome p450	(±)18-HETE	-1.21 (-2.33, -0.1)	-0.62 (-1.61, 0.38)	-1.21 (-2.96, 0.55)	-0.52 (-1.33, 0.29)	0.11 (-1, 1.22)	-0.83 (-1.89, 0.24)
Cytochrome p450	20-carboxy Arachidonic Acid	-0.34 (-0.89, 0.22)	-0.3 (-0.77, 0.18)	-0.35 (-1.2, 0.51)	-0.11 (-0.52, 0.31)	0.17 (-0.36, 0.71)	-0.39 (-0.9, 0.13)
Cytochrome p450	20(S)-HETE	-0.17 (-1.23, 0.89)	0.31 (-0.63, 1.25)	0.43 (-1.22, 2.08)	0.37 (-0.48, 1.22)	-0.49 (-1.5, 0.51)	0.33 (-0.67, 1.33)
Cytochrome p450	(±)5,6-DHET	-0.53 (-1.25, 0.2)	-0.45 (-1.08, 0.17)	-0.32 (-1.43, 0.78)	-0.16 (-0.72, 0.39)	0.25 (-0.44, 0.95)	-0.34 (-0.99, 0.32)
Cytochrome p450	5(6)-EET	0.19 (-0.4, 0.78)	-0.12 (-0.63, 0.39)	0.56 (-0.37, 1.48)	0.3 (-0.19, 0.79)	0.38 (-0.19, 0.95)	0.24 (-0.33, 0.8)
Cytochrome p450	(±)8,9-DHET	-0.52 (-1.5, 0.46)	-0.53 (-1.39, 0.33)	0.13 (-1.37, 1.63)	-0.09 (-0.85, 0.68)	0.55 (-0.38, 1.48)	-0.34 (-1.24, 0.57)
Cytochrome p450	8(9)-EET	0.04 (-0.96, 1.05)	0.15 (-0.72, 1.02)	0.13 (-1.43, 1.7)	0.02 (-0.75, 0.79)	0.06 (-0.92, 1.03)	0.38 (-0.58, 1.34)
Cytochrome p450	9(10)-EpOME	0.6 (-0.81, 2)	0.1 (-1.15, 1.36)	-0.91 (-3.04, 1.22)	0.18 (-0.9, 1.27)	-0.81 (-2.13, 0.52)	0.09 (-1.21, 1.39)
Cytochrome p450	9s-HODE	-1.7 (-3.33, -0.07)	-1.15 (-2.57, 0.27)	-2.16 (-4.74, 0.42)	-0.95 (-2.27, 0.37)	-1.41 (-3.01, 0.18)	-1.56 (-3.09, -0.04)
Cytochrome p450	(±)9,10-DiHOME	-2.28 (-3.4, -1.17)	-0.6 (-1.61, 0.42)	-1.66 (-3.43, 0.11)	-0.46 (-1.4, 0.48)	-0.63 (-1.74, 0.48)	-0.93 (-2, 0.14)

Lipoxygenase	Leukotriene B4	0.32 (-0.9, 1.55)	0.05 (-0.98, 1.09)	0.7 (-1.21, 2.61)	0.84 (-0.05, 1.74)	0.14 (-1.1, 1.39)	0.66 (-0.53, 1.85)
Lipoxygenase	Leukotriene D4	-0.79 (-1.68, 0.1)	-0.46 (-1.25, 0.33)	0.06 (-1.38, 1.5)	-0.05 (-0.8, 0.69)	-0.79 (-1.66, 0.09)	-0.79 (-1.66, 0.09)
Lipoxygenase	Leukotriene E4	0.72 (-0.05, 1.49)	-0.28 (-0.97, 0.41)	0.58 (-0.64, 1.79)	0.44 (-0.14, 1.02)	0.77 (0.03, 1.51)	0.41 (-0.34, 1.16)
Lipoxygenase	Resolvin D1	0.29 (-0.78, 1.37)	0.12 (-0.83, 1.06)	0.52 (-1.13, 2.18)	-0.02 (-0.85, 0.82)	0.1 (-0.93, 1.12)	0.37 (-0.64, 1.38)
Lipoxygenase	Resolvin D2	-0.26 (-1.7, 1.19)	0.22 (-1.02, 1.46)	1.24 (-1.01, 3.49)	0.62 (-0.47, 1.7)	1.46 (0.1, 2.82)	0.76 (-0.56, 2.08)
Lipoxygenase	12(S)-HETE	1.23 (-0.14, 2.6)	-0.53 (-1.89, 0.84)	1.8 (-0.3, 3.91)	0.51 (-0.51, 1.52)	0.89 (-0.41, 2.18)	0.61 (-0.84, 2.06)
Lipoxygenase	12-OxoETE	-0.29 (-1.25, 0.67)	-0.11 (-0.94, 0.71)	0.05 (-1.45, 1.56)	0.2 (-0.53, 0.92)	0.65 (-0.27, 1.58)	0.05 (-0.87, 0.96)
Lipoxygenase	13S-HODE	-1.21 (-2.71, 0.28)	-1 (-2.37, 0.37)	-0.03 (-2.35, 2.3)	-0.32 (-1.5, 0.86)	-0.39 (-1.83, 1.06)	-0.62 (-2.07, 0.83)
Lipoxygenase	13-OxoODE	0.06 (-1.43, 1.55)	0.89 (-0.4, 2.18)	-0.17 (-2.52, 2.18)	-0.44 (-1.59, 0.71)	0 (-1.43, 1.44)	0.16 (-1.27, 1.6)
Lipoxygenase	15(S)-HETE	-0.7 (-1.71, 0.3)	-0.53 (-1.41, 0.36)	0.02 (-1.55, 1.58)	-0.53 (-1.35, 0.28)	0.79 (-0.21, 1.79)	-0.44 (-1.42, 0.55)
Lipoxygenase	15-OxoETE	-0.05 (-1.02, 0.93)	-0.25 (-1.08, 0.58)	0.46 (-1.03, 1.95)	0.14 (-0.59, 0.87)	0.62 (-0.29, 1.53)	0.36 (-0.53, 1.25)
Lipoxygenase	5(S)-HETE	-0.41 (-1.58, 0.75)	-0.19 (-1.22, 0.83)	0.21 (-1.6, 2.02)	-0.28 (-1.19, 0.62)	1.07 (-0.07, 2.21)	0.01 (-1.13, 1.16)
Lipoxygenase	5-OxoETE	-0.11 (-0.86, 0.63)	-0.21 (-0.86, 0.44)	0.18 (-1.01, 1.37)	-0.02 (-0.61, 0.57)	0.45 (-0.28, 1.17)	-0.15 (-0.86, 0.57)
Lipoxygenase	8(S)-HETE	-0.88 (-1.81, 0.05)	-0.64 (-1.46, 0.17)	-0.28 (-1.73, 1.18)	-0.49 (-1.22, 0.24)	0.59 (-0.31, 1.5)	-0.54 (-1.42, 0.35)
Parent Compound	Arachidonic Acid	0.36 (-1.1, 1.82)	-0.44 (-1.72, 0.83)	0.34 (-1.94, 2.63)	-0.06 (-1.23, 1.11)	0.2 (-1.21, 1.6)	-0.16 (-1.54, 1.23)
Parent Compound	Docosahexaenoic Acid	-0.92 (-1.86, 0.03)	-0.73 (-1.54, 0.08)	-0.65 (-2.13, 0.82)	-0.47 (-1.18, 0.23)	0.22 (-0.71, 1.15)	-0.58 (-1.45, 0.3)
Parent Compound	Eicosapentaenoic Acid	-1.1 (-2.19, -0.02)	-0.66 (-1.59, 0.26)	-0.12 (-1.86, 1.62)	-0.54 (-1.4, 0.32)	0.32 (-0.74, 1.38)	-0.46 (-1.47, 0.55)
Parent Compound	Linoleic Acid	-0.58 (-1.56, 0.4)	-0.59 (-1.43, 0.26)	-0.29 (-1.83, 1.24)	-0.03 (-0.78, 0.71)	0.58 (-0.37, 1.52)	-0.37 (-1.27, 0.53)
Parent Compound	α-Linolenic Acid	-0.56 (-1.57, 0.45)	-0.55 (-1.43, 0.34)	-0.63 (-2.18, 0.93)	-0.07 (-0.81, 0.67)	-0.17 (-1.16, 0.81)	-0.61 (-1.55, 0.33)

**Male**							
Group Name	Bioactive Lipids	Adaptive	Cognitive	Communication	Motor	Personal-Social	Total
Cyclooxygenase	Bicyclo Prostaglandin E1	-0.08 (-2.46, 2.29)	-0.09 (-2.04, 1.86)	-2.37 (-5.46, 0.73)	-0.93 (-2.58, 0.72)	-1.07 (-2.94, 0.8)	-0.98 (-2.87, 0.9)
Cyclooxygenase	Bicyclo Prostaglandin E2	-0.89 (-3.11, 1.32)	-0.78 (-2.54, 0.99)	-3.11 (-5.86, -0.35)	-0.24 (-1.7, 1.21)	-0.14 (-1.91, 1.62)	-1.65 (-3.35, 0.06)
Cyclooxygenase	15-deoxy-Δ12,14-Prostaglandin J2	0.87 (-0.81, 2.55)	-0.04 (-1.45, 1.36)	2.71 (0.49, 4.92)	0.02 (-1.12, 1.17)	1.84 (0.55, 3.13)	0.48 (-0.95, 1.91)
Cyclooxygenase	13,14-dihydro-15-keto Prostaglandin D2	0.55 (-1.32, 2.43)	-0.72 (-2.38, 0.94)	0.95 (-1.59, 3.49)	0.65 (-0.6, 1.9)	0.91 (-0.59, 2.42)	0.12 (-1.61, 1.84)
Cyclooxygenase	13,14-dihydro-15-keto Prostaglandin E2	0.47 (-1.85, 2.79)	0.19 (-1.65, 2.04)	1.57 (-1.64, 4.79)	-0.13 (-1.66, 1.4)	0.41 (-1.49, 2.31)	0.26 (-1.62, 2.13)
Cyclooxygenase	13,14-dihydro-15-keto Prostaglandin F2α	-1.91 (-4.4, 0.59)	-0.94 (-3.09, 1.21)	-2.92 (-6.28, 0.44)	-0.57 (-2.41, 1.26)	-0.89 (-2.92, 1.14)	-1.15 (-3.25, 0.95)
Cyclooxygenase	13,14-dihydro-15-keto Prostaglandin J2	-0.39 (-1.91, 1.12)	-0.42 (-1.6, 0.75)	-0.3 (-2.33, 1.74)	-0.57 (-1.53, 0.39)	-0.5 (-1.7, 0.69)	-0.6 (-1.74, 0.54)
Cyclooxygenase	Prostaglandin A2	-0.57 (-4.16, 3.01)	1.56 (-1.36, 4.49)	-1.35 (-6.26, 3.56)	-0.19 (-3.76, 3.38)	-1.79 (-4.62, 1.03)	0.65 (-3.38, 4.68)
Cyclooxygenase	Prostaglandin B2	0.51 (-1.72, 2.74)	-0.58 (-2.35, 1.2)	1.74 (-1.28, 4.77)	0.1 (-1.29, 1.48)	2.58 (0.9, 4.27)	-0.42 (-2.38, 1.54)
Cyclooxygenase	Prostaglandin D2	-0.04 (-2.33, 2.24)	-2.19 (-4.05, -0.33)	-1.64 (-4.69, 1.41)	-0.19 (-1.52, 1.14)	1.03 (-0.79, 2.85)	-1.31 (-3.18, 0.56)
Cyclooxygenase	Prostaglandin D3	-0.18 (-1.88, 1.52)	-1.83 (-3.2, -0.45)	-1.7 (-3.81, 0.42)	0 (-1.01, 1.02)	0.37 (-0.93, 1.67)	-1.52 (-2.92, -0.13)
Cyclooxygenase	Prostaglandin E1 (power)	0.44 (-1.22, 2.1)	0.01 (-1.32, 1.34)	0.79 (-1.4, 2.97)	-0.09 (-1.26, 1.08)	-0.14 (-1.44, 1.16)	-0.22 (-1.68, 1.24)
Cyclooxygenase	Prostaglandin E2	-2.29 (-4.53, -0.05)	-2.15 (-3.95, -0.35)	-3.32 (-6.31, -0.33)	0.12 (-1.36, 1.6)	-0.71 (-2.6, 1.19)	-1.64 (-3.41, 0.12)
Cyclooxygenase	Prostaglandin E3	-1.15 (-3.55, 1.26)	-1.24 (-3, 0.51)	-3.54 (-6.4, -0.69)	-0.2 (-1.59, 1.18)	-0.8 (-2.6, 1)	-1.71 (-3.44, 0.02)
Cyclooxygenase	Prostaglandin J2	1.24 (-1.44, 3.92)	-0.36 (-2.64, 1.92)	1.64 (-2.01, 5.3)	-0.43 (-2.34, 1.48)	0.77 (-1.4, 2.94)	-0.6 (-2.88, 1.69)
Cyclooxygenase	Thromboxane B2	-0.11 (-1.8, 1.58)	-0.32 (-1.74, 1.1)	1.4 (-0.8, 3.6)	-0.02 (-1.04, 0.99)	0.73 (-0.59, 2.04)	0.53 (-0.82, 1.89)
Cyclooxygenase	9-OxoODE	-0.96 (-4.1, 2.18)	-0.89 (-3.38, 1.59)	1.03 (-3.2, 5.27)	-0.81 (-2.78, 1.15)	1.08 (-1.43, 3.58)	-0.81 (-3.2, 1.58)
Cytochrome p450	11(S)-HETE	-0.4 (-2.05, 1.26)	-0.52 (-1.83, 0.78)	0.45 (-1.77, 2.68)	-0.31 (-1.45, 0.84)	0.76 (-0.55, 2.07)	-0.37 (-1.7, 0.96)
Cytochrome p450	(±)11,12-DHET	-2.07 (-5.21, 1.07)	-0.4 (-2.98, 2.17)	-0.26 (-4.6, 4.08)	0.72 (-1.35, 2.79)	1.09 (-1.49, 3.67)	-0.16 (-2.64, 2.32)
Cytochrome p450	11(12)-EET	1.45 (-0.51, 3.42)	0.09 (-1.51, 1.68)	3.27 (0.69, 5.84)	-0.87 (-2.12, 0.38)	0.98 (-0.64, 2.59)	1.11 (-0.44, 2.66)
Cytochrome p450	12(13)-EpOME	0.64 (-1.15, 2.43)	1.21 (-0.23, 2.66)	0.78 (-1.64, 3.2)	0.66 (-0.48, 1.81)	-0.75 (-2.18, 0.67)	1.18 (-0.16, 2.51)

Cytochrome p450	(±)12,13-DiHOME	-4.14 (-7.07, -1.2)	0.98 (-1.58, 3.54)	-2.06 (-6.3, 2.18)	0.11 (-2.05, 2.27)	-1.64 (-4.19, 0.91)	-0.74 (-3.27, 1.79)
Cytochrome p450	14(15)-EET	-0.88 (-3.15, 1.39)	-1.05 (-2.87, 0.77)	1.47 (-1.58, 4.53)	-1.01 (-2.42, 0.4)	1.14 (-0.66, 2.94)	-0.57 (-2.35, 1.22)
Cytochrome p450	16(S)-HETE	-0.5 (-2.36, 1.36)	-0.7 (-2.13, 0.72)	-0.82 (-3.27, 1.64)	-0.15 (-1.41, 1.1)	0.99 (-0.46, 2.44)	-0.78 (-2.21, 0.64)
Cytochrome p450	17(S)-HETE	-1.62 (-3.4, 0.17)	-0.43 (-2.04, 1.19)	-2.33 (-4.63, -0.04)	-1.37 (-2.47, -0.28)	-0.77 (-2.18, 0.64)	-1.54 (-3.05, -0.03)
Cytochrome p450	(±)18-HETE	-1.72 (-3.55, 0.12)	-1.19 (-2.71, 0.33)	-2.74 (-5.12, -0.36)	-0.91 (-2, 0.17)	-0.62 (-2.11, 0.87)	-1.7 (-3.07, -0.32)
Cytochrome p450	20-carboxy Arachidonic Acid	-0.09 (-1.1, 0.92)	-0.33 (-1.09, 0.43)	-0.24 (-1.56, 1.08)	-0.24 (-0.85, 0.36)	0.26 (-0.53, 1.04)	-0.51 (-1.29, 0.26)
Cytochrome p450	20(S)-HETE	-0.13 (-2.32, 2.05)	2.01 (0.1, 3.93)	0.34 (-2.62, 3.3)	-0.3 (-1.76, 1.17)	-0.85 (-2.6, 0.89)	1.24 (-0.57, 3.06)
Cytochrome p450	(±)5,6-DHET	-0.49 (-1.83, 0.84)	-0.57 (-1.59, 0.45)	0.04 (-1.76, 1.83)	-0.2 (-1.05, 0.65)	0.42 (-0.64, 1.48)	-0.32 (-1.3, 0.65)
Cytochrome p450	5(6)-EET	0.69 (-0.36, 1.75)	0.02 (-0.84, 0.88)	1.86 (0.52, 3.2)	-0.05 (-0.82, 0.73)	0.9 (0.09, 1.72)	0.63 (-0.19, 1.46)
Cytochrome p450	(±)8,9-DHET	-0.26 (-2.08, 1.55)	-0.83 (-2.29, 0.63)	0.91 (-1.51, 3.34)	-0.01 (-1.2, 1.18)	1.21 (-0.2, 2.62)	-0.24 (-1.67, 1.19)
Cytochrome p450	8(9)-EET	1.41 (-0.3, 3.12)	1.11 (-0.29, 2.51)	2.34 (0.08, 4.61)	0.08 (-1.02, 1.18)	1.01 (-0.37, 2.38)	1.86 (0.6, 3.13)
Cytochrome p450	9(10)-EpOME	1.94 (-0.56, 4.45)	1.12 (-1, 3.24)	0.99 (-2.48, 4.47)	0.24 (-1.43, 1.9)	-0.78 (-2.82, 1.27)	1.27 (-0.7, 3.23)
Cytochrome p450	9s-HODE	-3.44 (-6.93, 0.05)	-1.32 (-4.19, 1.55)	-2.96 (-7.7, 1.77)	-1.36 (-3.69, 0.96)	-3.32 (-6.04, -0.59)	-2.37 (-5.03, 0.28)
Cytochrome p450	(±)9,10-DiHOME	-3.99 (-6.05, -1.93)	-0.36 (-2.3, 1.58)	-2.21 (-5.35, 0.94)	0.36 (-1.27, 1.99)	-0.65 (-2.57, 1.26)	-0.88 (-2.74, 0.98)
Lipoxygenase	Leukotriene B4	1.55 (-0.81, 3.91)	0.56 (-1.31, 2.42)	1.84 (-1.35, 5.04)	0.7 (-0.67, 2.07)	0.31 (-1.61, 2.23)	1.11 (-0.68, 2.89)
Lipoxygenase	Leukotriene D4	0.34 (-1.37, 2.05)	-0.92 (-2.36, 0.52)	1.32 (-0.99, 3.63)	0.48 (-0.73, 1.68)	-0.05 (-1.44, 1.33)	-0.06 (-1.52, 1.39)
Lipoxygenase	Leukotriene E4	1.57 (0.24, 2.9)	-0.44 (-1.62, 0.75)	2.24 (0.48, 4.01)	0.34 (-0.53, 1.21)	1.39 (0.35, 2.44)	0.87 (-0.23, 1.96)
Lipoxygenase	Resolvin D1	0.31 (-1.54, 2.16)	-0.47 (-1.99, 1.04)	0.72 (-1.77, 3.21)	0.22 (-1.08, 1.52)	0.15 (-1.33, 1.63)	0.17 (-1.3, 1.64)
Lipoxygenase	Resolvin D2	-0.59 (-3.5, 2.32)	-0.81 (-3.05, 1.43)	1.15 (-2.68, 4.98)	0.1 (-1.53, 1.73)	1.31 (-0.95, 3.57)	-0.41 (-2.58, 1.75)
Lipoxygenase	12(S)-HETE	2.59 (0.67, 4.51)	-0.94 (-3.01, 1.13)	2.72 (0.05, 5.4)	-0.37 (-1.76, 1.02)	0.77 (-0.87, 2.41)	0.28 (-1.71, 2.27)
Lipoxygenase	12-OxoETE	-0.37 (-2.19, 1.44)	-0.4 (-1.77, 0.97)	1.05 (-1.29, 3.39)	-0.11 (-1.2, 0.98)	0.68 (-0.7, 2.07)	-0.13 (-1.49, 1.23)
Lipoxygenase	13S-HODE	-1.46 (-4.58, 1.65)	-0.34 (-3.36, 2.68)	1.07 (-3.17, 5.31)	-1.18 (-3.12, 0.76)	-0.64 (-3.18, 1.91)	-0.86 (-3.7, 1.98)
Lipoxygenase	13-OxoODE	0.44 (-2.62, 3.49)	2.06 (-0.49, 4.6)	2.55 (-1.47, 6.56)	-0.67 (-2.72, 1.38)	0.24 (-2.19, 2.68)	1.53 (-0.91, 3.97)
Lipoxygenase	15(S)-HETE	-0.58 (-2.44, 1.27)	-1.05 (-2.53, 0.44)	-1.36 (-3.8, 1.07)	-0.78 (-2.08, 0.52)	0.68 (-0.83, 2.19)	-1.74 (-3.1, -0.39)
Lipoxygenase	15-OxoETE	1.24 (-0.46, 2.94)	0.56 (-0.8, 1.91)	2.54 (0.38, 4.7)	-0.01 (-1.11, 1.08)	1.17 (-0.14, 2.47)	1.39 (0.15, 2.62)
Lipoxygenase	5(S)-HETE	0.64 (-1.54, 2.82)	0.08 (-1.69, 1.84)	0.83 (-2.07, 3.74)	-0.61 (-2, 0.78)	0.65 (-1.07, 2.37)	-0.18 (-1.91, 1.55)
Lipoxygenase	5-OxoETE	0.91 (-0.45, 2.27)	0.1 (-1.04, 1.23)	1.79 (-0.01, 3.59)	-0.33 (-1.2, 0.54)	1.18 (0.12, 2.24)	0.36 (-0.73, 1.45)
Lipoxygenase	8(S)-HETE	-0.26 (-1.92, 1.39)	-0.51 (-1.88, 0.85)	0.03 (-2.21, 2.28)	-1.06 (-2.1, -0.03)	0.46 (-0.87, 1.8)	-0.82 (-2.1, 0.47)
Parent Compound	Arachidonic Acid	0.99 (-1.56, 3.54)	-1.03 (-3.11, 1.04)	0.66 (-2.85, 4.17)	-0.82 (-2.53, 0.89)	-0.31 (-2.37, 1.76)	-1.02 (-3.03, 0.99)
Parent Compound	Docosahexaenoic Acid	-1.04 (-2.6, 0.53)	-1.13 (-2.29, 0.03)	-1.55 (-3.58, 0.49)	-0.73 (-1.78, 0.31)	-0.36 (-1.58, 0.87)	-1.31 (-2.4, -0.22)
Parent Compound	Eicosapentaenoic Acid	-0.75 (-2.66, 1.16)	-1.28 (-2.73, 0.18)	-1.2 (-3.77, 1.38)	-1.19 (-2.53, 0.15)	-0.33 (-1.85, 1.19)	-1.57 (-2.91, -0.23)
Parent Compound	Linoleic Acid	-1.36 (-3.23, 0.51)	-1.73 (-3.16, -0.31)	-0.93 (-3.49, 1.63)	-0.4 (-1.55, 0.76)	0.1 (-1.43, 1.63)	-1.5 (-2.83, -0.18)
Parent Compound	α-Linolenic Acid	-1.14 (-2.77, 0.49)	-1.11 (-2.39, 0.17)	-1.43 (-3.62, 0.76)	-0.47 (-1.48, 0.53)	-0.67 (-1.98, 0.64)	-1.39 (-2.56, -0.23)

**Female**							
Group Name	Bioactive Lipids	Adaptive	Cognitive	Communication	Motor	Personal-Social	Total
Cyclooxygenase	Bicyclo Prostaglandin E1	0.09 (-1.64, 1.82)	0.77 (-0.9, 2.44)	-0.59 (-3.66, 2.48)	-1.5 (-2.95, -0.04)	0.01 (-1.91, 1.92)	-0.76 (-2.74, 1.21)
Cyclooxygenase	Bicyclo Prostaglandin E2	-0.55 (-2.1, 0.99)	-0.28 (-1.79, 1.23)	-1.58 (-4.32, 1.16)	-0.55 (-1.71, 0.6)	0.42 (-1.34, 2.17)	-0.56 (-2.32, 1.2)
Cyclooxygenase	15-deoxy-Δ12,14-Prostaglandin J2	-0.44 (-1.79, 0.91)	-0.38 (-1.64, 0.87)	-0.21 (-2.65, 2.23)	0.61 (-0.51, 1.74)	1.07 (-0.38, 2.52)	0.25 (-1.23, 1.73)
Cyclooxygenase	13,14-dihydro-15-keto Prostaglandin D2	0.1 (-1.02, 1.21)	-0.31 (-1.36, 0.74)	-0.79 (-2.78, 1.19)	0.44 (-0.53, 1.41)	-0.03 (-1.22, 1.17)	-0.09 (-1.33, 1.14)
Cyclooxygenase	13,14-dihydro-15-keto Prostaglandin E2	-0.46 (-1.99, 1.08)	-0.51 (-1.91, 0.88)	0.36 (-2.53, 3.25)	1.18 (-0.22, 2.59)	-0.13 (-1.82, 1.57)	0.02 (-1.68, 1.72)
Cyclooxygenase	13,14-dihydro-15-keto Prostaglandin F2α	-0.36 (-1.74, 1.01)	-0.23 (-1.54, 1.08)	0.69 (-1.85, 3.22)	-0.52 (-1.81, 0.77)	0.12 (-1.43, 1.66)	-0.32 (-1.89, 1.25)
Cyclooxygenase	13,14-dihydro-15-keto Prostaglandin J2	0.85 (-0.32, 2.01)	0.5 (-0.65, 1.65)	1.54 (-0.52, 3.6)	0.22 (-0.79, 1.23)	1.2 (-0.09, 2.5)	1 (-0.32, 2.32)

Cyclooxygenase	Prostaglandin A2	-1.08 (-3.53, 1.38)	0.16 (-2.11, 2.44)	-0.58 (-4.78, 3.62)	0.49 (-1.43, 2.42)	-0.69 (-3.26, 1.89)	-0.33 (-2.89, 2.23)
Cyclooxygenase	Prostaglandin B2	-0.19 (-1.15, 0.77)	-0.23 (-1.12, 0.66)	-0.36 (-2.14, 1.42)	0.27 (-0.62, 1.16)	0.42 (-0.65, 1.48)	-0.04 (-1.12, 1.05)
Cyclooxygenase	Prostaglandin D2	0.45 (-0.67, 1.56)	0.12 (-0.98, 1.21)	-1.1 (-3.05, 0.85)	-0.69 (-1.64, 0.25)	0.17 (-1.03, 1.38)	-0.31 (-1.59, 0.98)
Cyclooxygenase	Prostaglandin D3	-0.46 (-1.67, 0.76)	-1.17 (-2.29, -0.06)	-1.53 (-3.65, 0.58)	-1.15 (-2.11, -0.18)	0.26 (-1.03, 1.55)	-0.89 (-2.19, 0.41)
Cyclooxygenase	Prostaglandin E1 (power)	-0.47 (-1.61, 0.67)	-0.84 (-1.97, 0.29)	-0.21 (-2.23, 1.82)	0.74 (-0.27, 1.75)	0.13 (-1.17, 1.42)	-0.26 (-1.57, 1.05)
Cyclooxygenase	Prostaglandin E2	-0.77 (-2.35, 0.82)	-0.62 (-2.08, 0.83)	-0.57 (-3.46, 2.31)	-0.74 (-2.06, 0.58)	1.15 (-0.57, 2.87)	-0.32 (-2.05, 1.41)
Cyclooxygenase	Prostaglandin E3	-0.69 (-1.9, 0.53)	-1.05 (-2.14, 0.04)	-1.71 (-3.86, 0.44)	-1.78 (-2.74, -0.83)	-0.82 (-2.1, 0.46)	-1.66 (-2.88, -0.45)
Cyclooxygenase	Prostaglandin J2	-1.39 (-3.15, 0.38)	-1.23 (-2.82, 0.37)	-0.45 (-3.71, 2.81)	1.26 (-0.22, 2.73)	-0.53 (-2.47, 1.4)	-0.9 (-2.83, 1.02)
Cyclooxygenase	Thromboxane B2	-0.57 (-1.83, 0.69)	-0.2 (-1.45, 1.05)	-1.59 (-3.85, 0.68)	0.29 (-0.73, 1.31)	-0.2 (-1.64, 1.24)	-0.73 (-2.19, 0.72)
Cyclooxygenase	9-OxoODE	0.16 (-1.83, 2.14)	0.73 (-1.13, 2.59)	-1.41 (-4.85, 2.03)	-0.69 (-2.38, 0.99)	-0.74 (-2.89, 1.4)	-0.78 (-2.91, 1.35)
Cytochrome p450	11(S)-HETE	-0.66 (-1.89, 0.56)	0.02 (-1.18, 1.22)	-0.65 (-2.83, 1.53)	0.07 (-0.96, 1.11)	0.99 (-0.37, 2.35)	0.13 (-1.26, 1.52)
Cytochrome p450	(±)11,12-DHET	0.56 (-1.34, 2.47)	1.59 (-0.11, 3.28)	0.04 (-3.32, 3.39)	-0.46 (-2.13, 1.2)	0.29 (-1.74, 2.32)	0.51 (-1.51, 2.53)
Cytochrome p450	11(12)-EET	-0.96 (-2.61, 0.68)	-0.93 (-2.46, 0.61)	-1.72 (-4.75, 1.32)	0.28 (-1.21, 1.77)	-0.47 (-2.37, 1.42)	-0.57 (-2.5, 1.37)
Cytochrome p450	12(13)-EpOME	-0.2 (-1.41, 1.02)	-0.7 (-1.83, 0.43)	-0.94 (-3.06, 1.17)	0.21 (-0.89, 1.3)	-0.89 (-2.18, 0.39)	-0.47 (-1.78, 0.83)
Cytochrome p450	(±)12,13-DiHOME	-1.4 (-3.65, 0.85)	-2.38 (-4.41, -0.34)	-4.37 (-8.33, -0.41)	-1.08 (-3.14, 0.97)	-1.7 (-4.15, 0.75)	-2.72 (-5.13, -0.31)
Cytochrome p450	14(15)-EET	-1.48 (-3.19, 0.22)	-0.98 (-2.58, 0.62)	-2.05 (-5.2, 1.1)	0.5 (-1.09, 2.08)	-0.7 (-2.6, 1.2)	-1.14 (-3.03, 0.75)
Cytochrome p450	16(S)-HETE	-0.35 (-1.64, 0.95)	0.23 (-0.97, 1.42)	-0.12 (-2.37, 2.13)	0.49 (-0.53, 1.52)	0.87 (-0.53, 2.28)	0.26 (-1.15, 1.68)
Cytochrome p450	17(S)-HETE	-0.47 (-1.83, 0.88)	-0.15 (-1.45, 1.14)	0.1 (-2.22, 2.42)	-0.32 (-1.46, 0.81)	0.22 (-1.29, 1.73)	-0.37 (-1.88, 1.15)
Cytochrome p450	(±)18-HETE	-0.44 (-1.95, 1.07)	0.36 (-1.12, 1.85)	0.71 (-2.03, 3.44)	-0.11 (-1.37, 1.16)	0.7 (-1.05, 2.45)	0.01 (-1.75, 1.78)
Cytochrome p450	20-carboxy Arachidonic Acid	-0.27 (-0.94, 0.4)	-0.18 (-0.83, 0.46)	-0.35 (-1.52, 0.83)	0.07 (-0.51, 0.65)	0.26 (-0.48, 1)	-0.14 (-0.88, 0.6)
Cytochrome p450	20(S)-HETE	-0.59 (-1.77, 0.6)	-0.68 (-1.77, 0.4)	0.14 (-2.01, 2.29)	0.49 (-0.56, 1.55)	-1.08 (-2.32, 0.17)	-0.44 (-1.72, 0.83)
Cytochrome p450	(±)5,6-DHET	-0.23 (-1.08, 0.63)	-0.22 (-1.04, 0.6)	-0.43 (-1.89, 1.03)	-0.05 (-0.78, 0.69)	0.34 (-0.58, 1.26)	-0.14 (-1.07, 0.79)
Cytochrome p450	5(6)-EET	-0.03 (-0.71, 0.65)	-0.2 (-0.84, 0.43)	-0.26 (-1.48, 0.97)	0.69 (0.06, 1.32)	0.05 (-0.71, 0.82)	0.09 (-0.68, 0.86)
Cytochrome p450	(±)8,9-DHET	-0.05 (-1.23, 1.13)	-0.08 (-1.19, 1.04)	0 (-2.01, 2.01)	0.27 (-0.78, 1.31)	0.47 (-0.79, 1.73)	0.03 (-1.24, 1.3)
Cytochrome p450	8(9)-EET	-1.04 (-2.21, 0.12)	-0.61 (-1.69, 0.47)	-2.02 (-4.14, 0.1)	0.22 (-0.85, 1.28)	-1.16 (-2.46, 0.14)	-0.97 (-2.28, 0.35)
Cytochrome p450	9(10)-EpOME	-0.72 (-2.35, 0.91)	-0.87 (-2.4, 0.66)	-3 (-5.75, -0.26)	0 (-1.43, 1.43)	-1.43 (-3.14, 0.29)	-1.14 (-2.88, 0.59)
Cytochrome p450	9s-HODE	-0.93 (-2.73, 0.87)	-1.37 (-3.02, 0.29)	-1.96 (-5.24, 1.31)	-0.28 (-1.97, 1.42)	-1.05 (-3.04, 0.94)	-1.47 (-3.45, 0.51)
Cytochrome p450	(±)9,10-DiHOME	-1.14 (-2.51, 0.23)	-0.68 (-1.95, 0.59)	-1.23 (-3.63, 1.18)	-0.55 (-1.75, 0.65)	0.06 (-1.41, 1.53)	-0.67 (-2.12, 0.79)
Lipoxygenase	Leukotriene B4	-0.39 (-1.76, 0.97)	-0.55 (-1.79, 0.7)	-0.41 (-2.92, 2.11)	1.13 (-0.06, 2.32)	-0.7 (-2.33, 0.94)	-0.1 (-1.76, 1.55)
Lipoxygenase	Leukotriene D4	-1.26 (-2.23, -0.29)	-0.31 (-1.25, 0.63)	-0.96 (-2.85, 0.94)	-0.37 (-1.34, 0.59)	-1.21 (-2.32, -0.1)	-1.15 (-2.27, -0.03)
Lipoxygenase	Leukotriene E4	0 (-0.92, 0.92)	-0.19 (-1.05, 0.68)	-1.01 (-2.66, 0.63)	0.54 (-0.27, 1.35)	0.33 (-0.69, 1.35)	0.1 (-0.94, 1.15)
Lipoxygenase	Resolvin D1	0.24 (-1.07, 1.55)	0.94 (-0.28, 2.16)	0.28 (-2.02, 2.58)	-0.44 (-1.57, 0.68)	0.21 (-1.19, 1.61)	0.52 (-0.91, 1.94)
Lipoxygenase	Resolvin D2	0.16 (-1.46, 1.77)	0.44 (-1.04, 1.93)	1.06 (-1.88, 4)	1.39 (-0.07, 2.85)	1.94 (0.26, 3.62)	1.38 (-0.35, 3.11)
Lipoxygenase	12(S)-HETE	-0.61 (-2.6, 1.39)	-0.02 (-1.86, 1.81)	0.09 (-3.49, 3.68)	1.3 (-0.23, 2.83)	1.2 (-0.92, 3.33)	0.84 (-1.3, 2.98)
Lipoxygenase	12-OxoETE	0.06 (-1.05, 1.18)	0.19 (-0.88, 1.27)	-0.73 (-2.82, 1.35)	0.7 (-0.28, 1.68)	0.68 (-0.58, 1.94)	0.3 (-1, 1.6)
Lipoxygenase	13S-HODE	-0.53 (-2.21, 1.16)	-1.41 (-2.95, 0.14)	-0.53 (-3.45, 2.4)	0.36 (-1.13, 1.85)	-0.42 (-2.24, 1.41)	-0.67 (-2.48, 1.14)
Lipoxygenase	13-OxoODE	-0.06 (-1.67, 1.56)	0.92 (-0.55, 2.38)	-1.3 (-4.28, 1.68)	-0.5 (-1.9, 0.91)	0.05 (-1.74, 1.84)	-0.13 (-1.93, 1.66)
Lipoxygenase	15(S)-HETE	-0.34 (-1.56, 0.88)	0.07 (-1.07, 1.22)	1.03 (-1.08, 3.13)	0.04 (-1.07, 1.15)	1.2 (-0.16, 2.56)	0.56 (-0.81, 1.94)
Lipoxygenase	15-OxoETE	-1.01 (-2.17, 0.16)	-1.06 (-2.13, 0.01)	-1.5 (-3.62, 0.63)	0.54 (-0.47, 1.54)	-0.16 (-1.45, 1.12)	-0.54 (-1.83, 0.76)
Lipoxygenase	5(S)-HETE	-0.75 (-2.1, 0.6)	-0.26 (-1.57, 1.05)	-0.16 (-2.62, 2.3)	0.24 (-0.97, 1.44)	1.59 (0.07, 3.11)	0.4 (-1.2, 1.99)
Lipoxygenase	5-OxoETE	-0.67 (-1.52, 0.18)	-0.35 (-1.16, 0.45)	-1.16 (-2.78, 0.47)	0.36 (-0.44, 1.15)	-0.19 (-1.16, 0.78)	-0.44 (-1.41, 0.54)
Lipoxygenase	8(S)-HETE	-0.99 (-2.21, 0.23)	-0.33 (-1.52, 0.86)	0.04 (-2.12, 2.2)	0.36 (-0.73, 1.44)	1.05 (-0.3, 2.39)	0.06 (-1.31, 1.43)
Parent Compound	Arachidonic Acid	0.07 (-1.82, 1.96)	-0.34 (-2.1, 1.43)	-0.71 (-4.16, 2.74)	0.19 (-1.56, 1.93)	1.26 (-0.81, 3.32)	0.42 (-1.68, 2.53)
Parent Compound	Docosahexaenoic Acid	-0.35 (-1.56, 0.87)	0.11 (-1.09, 1.31)	0.36 (-1.74, 2.46)	-0.23 (-1.22, 0.75)	1.28 (-0.06, 2.61)	0.48 (-0.88, 1.84)
Parent Compound	Eicosapentaenoic Acid	-0.91 (-2.25, 0.43)	0.2 (-1.05, 1.45)	0.88 (-1.51, 3.26)	0.08 (-1.11, 1.27)	1.18 (-0.23, 2.59)	0.62 (-0.81, 2.05)
Parent Compound	Linoleic Acid	0.03 (-1.06, 1.11)	0.16 (-0.84, 1.17)	0.17 (-1.8, 2.15)	0.04 (-0.93, 1)	1.09 (-0.08, 2.26)	0.42 (-0.78, 1.62)
Parent Compound	α-Linolenic Acid	0.25 (-1.04, 1.54)	0.38 (-0.84, 1.6)	0.46 (-1.83, 2.74)	0.06 (-1.03, 1.15)	0.82 (-0.63, 2.27)	0.44 (-1.01, 1.9)

Supplementary Table S4. Percent change of CBCL/1.5-5 scores by doubling increase in maternal bioactive lipid concentration by child sex

**All**				
Group Name	Bioactive Lipids	Externalizing Score	Internalizing Score	Total Score
Cyclooxygenase	Bicyclo Prostaglandin E1	2.79 (-5.63, 11.97)	8.1 (-1.6, 18.75)	5.81 (-2.73, 15.1)
Cyclooxygenase	Bicyclo Prostaglandin E2	-0.49 (-5.46, 4.73)	0.53 (-6.13, 7.66)	1.51 (-4.12, 7.48)
Cyclooxygenase	15-deoxy-Δ12,14-Prostaglandin J2	-0.38 (-6.08, 5.67)	-5.41 (-11.95, 1.61)	-1.07 (-6.24, 4.38)
Cyclooxygenase	13,14-dihydro-15-keto Prostaglandin D2	-3.77 (-10.53, 3.5)	-3.86 (-11.8, 4.79)	-4.19 (-10.48, 2.54)
Cyclooxygenase	13,14-dihydro-15-keto Prostaglandin E2	-3.61 (-11.51, 5)	-5.11 (-13.96, 4.65)	-3.37 (-10.81, 4.68)
Cyclooxygenase	13,14-dihydro-15-keto Prostaglandin F2α	-0.88 (-8.58, 7.46)	-4.78 (-14.13, 5.6)	0.7 (-7.77, 9.94)
Cyclooxygenase	13,14-dihydro-15-keto Prostaglandin J2	2.62 (-3.82, 9.5)	-1.03 (-8.17, 6.66)	2.3 (-3.93, 8.93)
Cyclooxygenase	Prostaglandin A2	12.69 (1.67, 24.9)	4.85 (-8.01, 19.51)	13.55 (2.22, 26.14)
Cyclooxygenase	Prostaglandin B2	-1.13 (-6.91, 5)	-5.36 (-11.53, 1.25)	0.12 (-5.52, 6.1)
Cyclooxygenase	Prostaglandin D2	-0.05 (-5.91, 6.18)	0.16 (-6.71, 7.54)	1.85 (-4.01, 8.07)
Cyclooxygenase	Prostaglandin D3	0.46 (-5.79, 7.12)	-1.09 (-8.55, 6.99)	1.81 (-4.53, 8.57)
Cyclooxygenase	Prostaglandin E1 (power)	3.68 (-2.76, 10.56)	-4.23 (-10.68, 2.68)	3.49 (-2.67, 10.05)
Cyclooxygenase	Prostaglandin E2	-2.88 (-10.33, 5.19)	-3.91 (-12.82, 5.9)	-0.46 (-8.28, 8.04)
Cyclooxygenase	Prostaglandin E3	-0.36 (-6.69, 6.4)	2.77 (-5.47, 11.73)	0.8 (-6.17, 8.29)
Cyclooxygenase	Prostaglandin J2	10.82 (1.38, 21.15)	-0.28 (-10.73, 11.38)	8.94 (-0.96, 19.84)
Cyclooxygenase	Thromboxane B2	-0.64 (-7.02, 6.18)	-4.37 (-11.7, 3.58)	0.68 (-5.41, 7.17)
Cyclooxygenase	9-OxoODE	12.5 (0.9, 25.44)	0.28 (-12.12, 14.44)	15.73 (3.32, 29.63)
Cytochrome p450	11(S)-HETE	0.68 (-5.65, 7.44)	-4.11 (-10.99, 3.31)	1.77 (-4.56, 8.53)
Cytochrome p450	(±)11,12-DHET	-2.58 (-12.51, 8.47)	-3.78 (-14.86, 8.75)	-2.07 (-11.81, 8.76)
Cytochrome p450	11(12)-EET	4.73 (-2.07, 11.99)	-5.75 (-13.74, 2.99)	3.08 (-3.6, 10.23)
Cytochrome p450	12(13)-EpOME	8.27 (1.72, 15.23)	6.47 (-1.6, 15.21)	9.87 (3.17, 17)
Cytochrome p450	(±)12,13-DiHOME	8.95 (-0.98, 19.87)	2.27 (-8.99, 14.93)	11.93 (1.48, 23.46)
Cytochrome p450	14(15)-EET	3.38 (-4.7, 12.14)	-5.39 (-14.44, 4.62)	3.69 (-4.88, 13.03)
Cytochrome p450	16(S)-HETE	1.92 (-4.67, 8.96)	-5.37 (-12.4, 2.22)	3.73 (-3.4, 11.38)
Cytochrome p450	17(S)-HETE	0.19 (-6.91, 7.84)	-5.85 (-14.14, 3.24)	2.79 (-5.13, 11.36)
Cytochrome p450	(±)18-HETE	0.5 (-6.76, 8.33)	-1.56 (-10.59, 8.39)	3.84 (-4.11, 12.44)
Cytochrome p450	20-carboxy Arachidonic Acid	3.36 (-0.94, 7.84)	-3.39 (-8.28, 1.76)	4.31 (-0.53, 9.39)
Cytochrome p450	20(S)-HETE	12.38 (2.7, 22.97)	6.22 (-4.58, 18.23)	14.71 (4.6, 25.8)
Cytochrome p450	(±)5,6-DHET	-0.57 (-5.46, 4.58)	-6.86 (-12.4, -0.97)	0.01 (-4.84, 5.1)
Cytochrome p450	5(6)-EET	-0.9 (-5.01, 3.39)	-7.43 (-11.95, -2.67)	-1.49 (-5.62, 2.82)
Cytochrome p450	(±)8,9-DHET	1.63 (-5.14, 8.89)	-6.78 (-14.09, 1.15)	1.84 (-4.81, 8.97)
Cytochrome p450	8(9)-EET	3.76 (-2.82, 10.8)	0.35 (-6.59, 7.8)	3.81 (-2.6, 10.65)
Cytochrome p450	9(10)-EpOME	9.91 (-0.04, 20.86)	5.4 (-5.51, 17.55)	11.41 (1.5, 22.28)
Cytochrome p450	9s-HODE	10.75 (-1.06, 23.97)	1.03 (-12.18, 16.23)	13.84 (1.55, 27.61)
Cytochrome p450	(±)9,10-DiHOME	7.69 (0.29, 15.64)	-3.11 (-11.72, 6.35)	8.59 (0.27, 17.61)
Lipoxygenase	Leukotriene B4	-2.44 (-8.2, 3.69)	-1.58 (-8.92, 6.36)	-3.18 (-9.14, 3.18)
Lipoxygenase	Leukotriene D4	3.76 (-2.44, 10.37)	1.4 (-5.8, 9.15)	6.61 (0.03, 13.63)
Lipoxygenase	Leukotriene E4	-3.1 (-7.23, 1.21)	-6.06 (-11.39, -0.41)	-4 (-8.39, 0.6)
Lipoxygenase	Resolvin D1	8.61 (1.31, 16.43)	8.13 (-0.89, 17.97)	10.85 (2.78, 19.55)
Lipoxygenase	Resolvin D2	10 (-0.27, 21.32)	0.32 (-11.43, 13.64)	14.15 (3, 26.5)

Lipoxygenase	12(S)-HETE	0.2 (-7.23, 8.22)	0.77 (-8.82, 11.38)	4.98 (-2.75, 13.33)
Lipoxygenase	12-OxoETE	2.96 (-3.49, 9.84)	-1.94 (-9.38, 6.12)	4.99 (-2.19, 12.68)
Lipoxygenase	13S-HODE	12.61 (2.31, 23.94)	-0.5 (-11.64, 12.06)	16.8 (6.16, 28.52)
Lipoxygenase	13-OxoODE	5.94 (-2.24, 14.8)	0.75 (-8.87, 11.38)	11.45 (2.67, 20.97)
Lipoxygenase	15(S)-HETE	-1.02 (-7.16, 5.51)	-4.47 (-12.18, 3.91)	-0.04 (-6.7, 7.1)
Lipoxygenase	15-OxoETE	5.09 (-0.15, 10.6)	-0.8 (-6.53, 5.27)	4.92 (-0.14, 10.24)
Lipoxygenase	5(S)-HETE	2.82 (-3.26, 9.28)	-2.73 (-9.64, 4.72)	3.44 (-2.51, 9.76)
Lipoxygenase	5-OxoETE	1.66 (-3.89, 7.54)	-5.9 (-11.76, 0.34)	2.86 (-2.65, 8.68)
Lipoxygenase	8(S)-HETE	-1.98 (-8.14, 4.59)	-5.84 (-12.49, 1.33)	-1.69 (-7.45, 4.43)
Parent Compound	Arachidonic Acid	2.92 (-5.16, 11.68)	1.07 (-9.02, 12.28)	8.03 (-0.64, 17.47)
Parent Compound	Docosahexaenoic Acid	3.28 (-3.4, 10.43)	-2.19 (-11.05, 7.55)	5.38 (-2.02, 13.35)
Parent Compound	Eicosapentaenoic Acid	0.12 (-7.65, 8.55)	-6.11 (-14.85, 3.53)	0.11 (-7.6, 8.47)
Parent Compound	Linoleic Acid	8.01 (1.01, 15.49)	-1.21 (-9.35, 7.67)	11.45 (2.99, 20.61)
Parent Compound	α-Linolenic Acid	10.93 (3.27, 19.16)	1.75 (-7.09, 11.44)	13.2 (5.11, 21.91)

**Male**				
Group Name	Bioactive Lipids	Externalizing Score	Internalizing Score	Total Score
Cyclooxygenase	Bicyclo Prostaglandin E1	2.19 (-10.11, 16.17)	9.11 (-6.23, 26.95)	7.04 (-6.06, 21.96)
Cyclooxygenase	Bicyclo Prostaglandin E2	-1.2 (-8.21, 6.34)	-5.26 (-13.71, 4.01)	-0.73 (-7.82, 6.9)
Cyclooxygenase	15-deoxy-Δ12,14-Prostaglandin J2	-3.41 (-10.88, 4.68)	-6.34 (-14.54, 2.66)	-3.19 (-10.52, 4.74)
Cyclooxygenase	13,14-dihydro-15-keto Prostaglandin D2	-5.95 (-13.73, 2.53)	-3.72 (-13.5, 7.17)	-5.2 (-13.22, 3.56)
Cyclooxygenase	13,14-dihydro-15-keto Prostaglandin E2	-8.37 (-17.5, 1.77)	-3.49 (-14.39, 8.79)	-7.69 (-16.96, 2.6)
Cyclooxygenase	13,14-dihydro-15-keto Prostaglandin F2α	-0.04 (-11.24, 12.59)	-3.4 (-14.74, 9.46)	1.68 (-9.99, 14.87)
Cyclooxygenase	13,14-dihydro-15-keto Prostaglandin J2	-3.09 (-10.76, 5.24)	-3.91 (-14.13, 7.53)	-2.64 (-11.38, 6.97)
Cyclooxygenase	Prostaglandin A2	12.96 (-4.18, 33.16)	8.29 (-10.07, 30.41)	16.04 (-1.61, 36.84)
Cyclooxygenase	Prostaglandin B2	-2.62 (-11.08, 6.63)	-7.58 (-16.7, 2.54)	-0.62 (-9.29, 8.87)
Cyclooxygenase	Prostaglandin D2	-5.3 (-13.27, 3.41)	-4.89 (-15.08, 6.53)	-3.07 (-11.85, 6.59)
Cyclooxygenase	Prostaglandin D3	-3.76 (-11.4, 4.54)	-4.79 (-13.64, 4.96)	-1.08 (-9.39, 7.99)
Cyclooxygenase	Prostaglandin E1 (power)	-0.41 (-8.74, 8.68)	-5.64 (-15.07, 4.84)	-1.13 (-9.23, 7.7)
Cyclooxygenase	Prostaglandin E2	-8.31 (-17.35, 1.71)	-2.4 (-14.82, 11.83)	-3.33 (-13.67, 8.26)
Cyclooxygenase	Prostaglandin E3	-1.43 (-11.91, 10.3)	0.06 (-12.43, 14.33)	0.57 (-9.74, 12.05)
Cyclooxygenase	Prostaglandin J2	11.52 (-3.06, 28.3)	3.43 (-12.53, 22.29)	15.82 (0.49, 33.49)
Cyclooxygenase	Thromboxane B2	-3.92 (-11.73, 4.59)	-8.33 (-17.96, 2.41)	-3.13 (-11.67, 6.23)
Cyclooxygenase	9-OxoODE	13.74 (-4.63, 35.65)	-1.7 (-18.66, 18.8)	15.1 (-4.45, 38.65)
Cytochrome p450	11(S)-HETE	-7.48 (-15.73, 1.57)	-10.48 (-19.68, -0.24)	-7.66 (-15.41, 0.81)
Cytochrome p450	(±)11,12-DHET	-8.82 (-22.12, 6.74)	-7.57 (-24.6, 13.3)	-10.17 (-23.24, 5.12)
Cytochrome p450	11(12)-EET	1.22 (-8.07, 11.45)	-9.04 (-18.95, 2.08)	0.02 (-9.31, 10.33)
Cytochrome p450	12(13)-EpOME	12.41 (1.74, 24.19)	7.33 (-5.07, 21.34)	14.54 (4.06, 26.08)
Cytochrome p450	(±)12,13-DiHOME	6.52 (-5.89, 20.57)	7.03 (-7.55, 23.91)	8.67 (-5.21, 24.58)
Cytochrome p450	14(15)-EET	3.49 (-7.98, 16.4)	-5.38 (-17.43, 8.43)	3.35 (-7.9, 15.97)
Cytochrome p450	16(S)-HETE	0.81 (-9.59, 12.42)	-7.04 (-16.72, 3.77)	3.16 (-7.91, 15.57)
Cytochrome p450	17(S)-HETE	1.7 (-10.23, 15.23)	-0.98 (-13.87, 13.84)	5.06 (-7.18, 18.92)
Cytochrome p450	(±)18-HETE	1.33 (-8.48, 12.18)	1.05 (-10.72, 14.37)	-0.11 (-9.42, 10.16)
Cytochrome p450	20-carboxy Arachidonic Acid	0.19 (-6.58, 7.45)	-4.84 (-12.09, 3.01)	0.45 (-6.3, 7.69)

Cytochrome p450	20(S)-HETE	19.04 (4.04, 36.21)	12 (-3.66, 30.21)	25.2 (10.39, 42)
Cytochrome p450	(±)5,6-DHET	-6.61 (-13.41, 0.73)	-9.92 (-17.22, -1.97)	-5.67 (-12.25, 1.4)
Cytochrome p450	5(6)-EET	-3.66 (-9.4, 2.44)	-8.53 (-14.92, -1.67)	-4.04 (-10.04, 2.37)
Cytochrome p450	(±)8,9-DHET	-4.29 (-13.41, 5.8)	-11.98 (-21.38, -1.46)	-5.09 (-13.9, 4.62)
Cytochrome p450	8(9)-EET	6.19 (-2.45, 15.6)	-0.19 (-9.76, 10.39)	5.65 (-3.68, 15.89)
Cytochrome p450	9(10)-EpOME	8.14 (-6.21, 24.67)	4.02 (-12.29, 23.35)	11.81 (-2.47, 28.18)
Cytochrome p450	9s-HODE	11.91 (-5.46, 32.47)	7.34 (-11.71, 30.5)	13.86 (-4.48, 35.72)
Cytochrome p450	(±)9,10-DiHOME	8.41 (-2.96, 21.11)	0.92 (-11.65, 15.28)	8.33 (-3.88, 22.1)
Lipoxygenase	Leukotriene B4	-1.99 (-10.14, 6.91)	-1.21 (-10.87, 9.48)	-1.2 (-8.9, 7.17)
Lipoxygenase	Leukotriene D4	3.69 (-5.76, 14.1)	0.84 (-9.49, 12.35)	7.5 (-2.86, 18.97)
Lipoxygenase	Leukotriene E4	-8.33 (-14.48, -1.73)	-13.33 (-20.17, -5.91)	-9.8 (-14.93, -4.36)
Lipoxygenase	Resolvin D1	8.19 (-2.47, 20.01)	3.14 (-8.74, 16.58)	9.36 (-1.6, 21.54)
Lipoxygenase	Resolvin D2	4.19 (-9.34, 19.74)	1.53 (-14.55, 20.63)	6.71 (-8.37, 24.26)
Lipoxygenase	12(S)-HETE	1.68 (-10.44, 15.43)	1.93 (-11.75, 17.75)	5.78 (-6.75, 20)
Lipoxygenase	12-OxoETE	-0.52 (-9.58, 9.44)	-1.66 (-12.81, 10.93)	2.42 (-8.04, 14.08)
Lipoxygenase	13S-HODE	15.77 (-0.04, 34.07)	2.66 (-13.41, 21.72)	16.17 (-0.3, 35.35)
Lipoxygenase	13-OxoODE	4.25 (-9.08, 19.52)	-7.91 (-21.39, 7.88)	7.27 (-8.08, 25.18)
Lipoxygenase	15(S)-HETE	-8.55 (-16.93, 0.67)	-5.68 (-16.73, 6.83)	-7.45 (-16.24, 2.26)
Lipoxygenase	15-OxoETE	6.14 (-2.3, 15.32)	0.64 (-8.46, 10.65)	6.64 (-1.8, 15.8)
Lipoxygenase	5(S)-HETE	-3.13 (-10.55, 4.9)	-5.54 (-14.76, 4.68)	-0.9 (-9.2, 8.16)
Lipoxygenase	5-OxoETE	-0.22 (-8.64, 8.98)	-3.37 (-12.93, 7.24)	2.83 (-6.74, 13.39)
Lipoxygenase	8(S)-HETE	-6.34 (-14.2, 2.24)	-2.67 (-12.72, 8.54)	-5.12 (-13.11, 3.61)
Parent Compound	Arachidonic Acid	-1.71 (-13, 11.04)	-2.21 (-15.23, 12.81)	2.67 (-9.39, 16.33)
Parent Compound	Docosahexaenoic Acid	-0.6 (-10.26, 10.09)	-4.6 (-15.21, 7.33)	0.65 (-9.18, 11.53)
Parent Compound	Eicosapentaenoic Acid	-5.69 (-15.53, 5.3)	-8.03 (-19.38, 4.92)	-5.03 (-15.48, 6.71)
Parent Compound	Linoleic Acid	10.22 (-0.91, 22.59)	2.32 (-9.68, 15.91)	13.05 (0.46, 27.22)
Parent Compound	α-Linolenic Acid	11.26 (0.5, 23.18)	5.72 (-6.25, 19.23)	13.9 (2.19, 26.95)

**Female**				
Group Name	Bioactive Lipids	Externalizing Score	Internalizing Score	Total Score
Cyclooxygenase	Bicyclo Prostaglandin E1	2.98 (-6.63, 13.59)	6.29 (-6.31, 20.58)	6.29 (-4.38, 18.16)
Cyclooxygenase	Bicyclo Prostaglandin E2	3.2 (-5.73, 12.99)	9.54 (-0.92, 21.11)	4.62 (-3.93, 13.92)
Cyclooxygenase	15-deoxy-Δ12,14-Prostaglandin J2	3.34 (-4.53, 11.86)	-7.21 (-16.37, 2.96)	2.86 (-5.54, 12.01)
Cyclooxygenase	13,14-dihydro-15-keto Prostaglandin D2	-3.05 (-13.2, 8.3)	-5.15 (-17.74, 9.37)	-3.12 (-13.56, 8.59)
Cyclooxygenase	13,14-dihydro-15-keto Prostaglandin E2	3.19 (-9.38, 17.51)	-4.84 (-20.17, 13.42)	2.93 (-10.33, 18.15)
Cyclooxygenase	13,14-dihydro-15-keto Prostaglandin F2α	-1.07 (-12.84, 12.29)	-11.51 (-23.2, 1.95)	-0.63 (-12.36, 12.67)
Cyclooxygenase	13,14-dihydro-15-keto Prostaglandin J2	8.04 (-1.97, 19.06)	2.79 (-7.62, 14.38)	8.4 (-1.25, 18.99)
Cyclooxygenase	Prostaglandin A2	7.32 (-6.65, 23.37)	4.75 (-11.83, 24.45)	10.84 (-3.22, 26.94)
Cyclooxygenase	Prostaglandin B2	-0.86 (-8.28, 7.16)	-5.61 (-13.76, 3.3)	-0.54 (-7.65, 7.12)
Cyclooxygenase	Prostaglandin D2	4.39 (-4.62, 14.25)	5.59 (-4.68, 16.97)	7.72 (-1.35, 17.62)
Cyclooxygenase	Prostaglandin D3	6.08 (-3.15, 16.18)	3.89 (-6.97, 16.02)	5.56 (-3.38, 15.32)
Cyclooxygenase	Prostaglandin E1 (power)	7.72 (-1.69, 18.03)	-2.05 (-11.62, 8.55)	7.48 (-1.32, 17.06)
Cyclooxygenase	Prostaglandin E2	1.35 (-8.9, 12.75)	-5.22 (-17.8, 9.3)	1.68 (-8.8, 13.36)

Cyclooxygenase	Prostaglandin E3	1.11 (-7.45, 10.46)	5.9 (-5.08, 18.14)	0.7 (-7.49, 9.62)
Cyclooxygenase	Prostaglandin J2	10.05 (-2.13, 23.75)	-4.79 (-17.23, 9.52)	8.21 (-4.12, 22.12)
Cyclooxygenase	Thromboxane B2	2.45 (-6.19, 11.89)	-0.49 (-10.68, 10.86)	5.8 (-3.33, 15.78)
Cyclooxygenase	9-OxoODE	13.77 (-1.46, 31.36)	2.54 (-13.4, 21.42)	18.68 (2.94, 36.82)
Cytochrome p450	11(S)-HETE	8.31 (-0.39, 17.76)	1.4 (-8.66, 12.56)	9.52 (0.7, 19.11)
Cytochrome p450	(±)11,12-DHET	1.14 (-11.32, 15.35)	2.87 (-11.59, 19.7)	2.65 (-9.75, 16.75)
Cytochrome p450	11(12)-EET	7.35 (-4.77, 21.02)	-0.38 (-14.08, 15.5)	8.37 (-3.26, 21.4)
Cytochrome p450	12(13)-EpOME	5.04 (-4.23, 15.19)	4.3 (-6.37, 16.18)	6.31 (-3.2, 16.75)
Cytochrome p450	(±)12,13-DiHOME	12.16 (-3.97, 31)	0.68 (-15.86, 20.46)	18.5 (2.18, 37.42)
Cytochrome p450	14(15)-EET	2.34 (-9.65, 15.92)	-6.13 (-19.42, 9.36)	4.62 (-6.65, 17.26)
Cytochrome p450	16(S)-HETE	2.43 (-6.84, 12.62)	-5.15 (-14.79, 5.58)	3.98 (-4.97, 13.78)
Cytochrome p450	17(S)-HETE	-0.04 (-11.41, 12.78)	-9.19 (-20.46, 3.68)	1.6 (-9.66, 14.26)
Cytochrome p450	(±)18-HETE	0.35 (-10.42, 12.42)	-5.48 (-17.7, 8.56)	4.93 (-6.96, 18.34)
Cytochrome p450	20-carboxy Arachidonic Acid	7.97 (1.45, 14.92)	-0.8 (-7.31, 6.16)	7.66 (1.45, 14.25)
Cytochrome p450	20(S)-HETE	5.05 (-7.2, 18.92)	2.24 (-11.48, 18.08)	7.62 (-5.14, 22.1)
Cytochrome p450	(±)5,6-DHET	4.84 (-1.65, 11.75)	-4.76 (-11.7, 2.72)	4.32 (-2.16, 11.23)
Cytochrome p450	5(6)-EET	1.49 (-4.73, 8.12)	-6.44 (-12.86, 0.44)	1.2 (-4.45, 7.18)
Cytochrome p450	(±)8,9-DHET	8.16 (-0.82, 17.95)	-3.62 (-13.83, 7.79)	8.36 (-0.54, 18.06)
Cytochrome p450	8(9)-EET	0.48 (-9, 10.95)	0.86 (-9.23, 12.07)	0.82 (-8.16, 10.67)
Cytochrome p450	9(10)-EpOME	11.4 (-2.13, 26.8)	4.74 (-9.79, 21.62)	12.76 (-0.21, 27.41)
Cytochrome p450	9s-HODE	14.66 (-0.33, 31.89)	-2.02 (-18.84, 18.28)	14.73 (-1.03, 33)
Cytochrome p450	(±)9,10-DiHOME	7.25 (-3.33, 18.98)	-8.97 (-19.22, 2.59)	9.53 (-1.83, 22.19)
Lipoxygenase	Leukotriene B4	-4.04 (-12.75, 5.55)	-2.76 (-13.28, 9.04)	-5.83 (-14.56, 3.79)
Lipoxygenase	Leukotriene D4	3.86 (-4.67, 13.16)	1.28 (-8.59, 12.21)	5.38 (-3.39, 14.95)
Lipoxygenase	Leukotriene E4	2.05 (-4.68, 9.24)	1.09 (-6.9, 9.78)	1.73 (-4.84, 8.74)
Lipoxygenase	Resolvin D1	9.69 (-0.93, 21.45)	17.05 (3.66, 32.18)	16.53 (5.79, 28.36)
Lipoxygenase	Resolvin D2	15.43 (-0.17, 33.47)	-0.61 (-17.77, 20.12)	17.29 (0.95, 36.27)
Lipoxygenase	12(S)-HETE	-1.57 (-11.86, 9.92)	-2.7 (-14.37, 10.56)	2.96 (-7.62, 14.75)
Lipoxygenase	12-OxoETE	6.52 (-2.52, 16.39)	-1.55 (-11.15, 9.09)	7.99 (-1.26, 18.1)
Lipoxygenase	13S-HODE	12.95 (0.13, 27.42)	-4.83 (-17.61, 9.92)	17.15 (4.06, 31.89)
Lipoxygenase	13-OxoODE	7.64 (-2.93, 19.37)	7.25 (-5.51, 21.72)	13.44 (2.33, 25.75)
Lipoxygenase	15(S)-HETE	6.94 (-2.64, 17.45)	-2.56 (-13.49, 9.74)	6.84 (-2.98, 17.66)
Lipoxygenase	15-OxoETE	4.61 (-1.78, 11.42)	-0.92 (-8.16, 6.89)	4.58 (-1.6, 11.14)
Lipoxygenase	5(S)-HETE	8.65 (-0.1, 18.18)	0.48 (-9.16, 11.15)	9.35 (0.54, 18.93)
Lipoxygenase	5-OxoETE	4.02 (-3.73, 12.41)	-6.56 (-14.72, 2.39)	4.3 (-3.45, 12.67)
Lipoxygenase	8(S)-HETE	2.82 (-6.27, 12.78)	-8.35 (-17.49, 1.8)	3.3 (-5.71, 13.18)
Parent Compound	Arachidonic Acid	8.41 (-4.45, 23)	4.44 (-11.1, 22.69)	9.1 (-4.43, 24.53)
Parent Compound	Docosahexaenoic Acid	9.55 (-0.79, 20.96)	-3.33 (-14.72, 9.58)	10.72 (-0.11, 22.72)
Parent Compound	Eicosapentaenoic Acid	5.06 (-6.84, 18.47)	-7.55 (-19.15, 5.71)	5.94 (-5.16, 18.33)
Parent Compound	Linoleic Acid	9.89 (-0.94, 21.92)	-4.2 (-14.54, 7.39)	10.51 (-0.55, 22.79)
Parent Compound	α-Linolenic Acid	9.5 (-2.54, 23.02)	-1.52 (-13.46, 12.08)	12.08 (0.23, 25.33)
